# A beginner’s guide on the use of brain organoids for neuroscientists: a systematic review

**DOI:** 10.1186/s13287-023-03302-x

**Published:** 2023-04-15

**Authors:** Lance A. Mulder, Josse A. Depla, Adithya Sridhar, Katja Wolthers, Dasja Pajkrt, Renata Vieira de Sá

**Affiliations:** 1 Department of Paediatric Infectious Diseases, Amsterdam UMC Location University of Amsterdam, Amsterdam, The Netherlands; 2 Department Medical Microbiology, OrganoVIR Labs, Amsterdam UMC Location University of Amsterdam, Amsterdam, the Netherlands; 3 Amsterdam Institute for Infection and Immunity, Infectious Diseases, Amsterdam, the Netherlands; 4 uniQure Biopharma B.V., Amsterdam, The Netherlands

**Keywords:** Human brain organoids, Neurodevelopment, Pluripotent stem cells, Cell type characterisation

## Abstract

**Background:**

The first human brain organoid protocol was presented in the beginning of the previous decade, and since then, the field witnessed the development of many new brain region-specific models, and subsequent protocol adaptations and modifications. The vast amount of data available on brain organoid technology may be overwhelming for scientists new to the field and consequently decrease its accessibility. Here, we aimed at providing a practical guide for new researchers in the field by systematically reviewing human brain organoid publications.

**Methods:**

Articles published between 2010 and 2020 were selected and categorised for brain organoid applications. Those describing neurodevelopmental studies or protocols for novel organoid models were further analysed for culture duration of the brain organoids, protocol comparisons of key aspects of organoid generation, and performed functional characterisation assays. We then summarised the approaches taken for different models and analysed the application of small molecules and growth factors used to achieve organoid regionalisation. Finally, we analysed articles for organoid cell type compositions, the reported time points per cell type, and for immunofluorescence markers used to characterise different cell types.

**Results:**

Calcium imaging and patch clamp analysis were the most frequently used neuronal activity assays in brain organoids. Neural activity was shown in all analysed models, yet network activity was age, model, and assay dependent. Induction of dorsal forebrain organoids was primarily achieved through combined (dual) SMAD and Wnt signalling inhibition. Ventral forebrain organoid induction was performed with dual SMAD and Wnt signalling inhibition, together with additional activation of the Shh pathway. Cerebral organoids and dorsal forebrain model presented the most cell types between days 35 and 60. At 84 days, dorsal forebrain organoids contain astrocytes and potentially oligodendrocytes. Immunofluorescence analysis showed cell type-specific application of non-exclusive markers for multiple cell types.

**Conclusions:**

We provide an easily accessible overview of human brain organoid cultures, which may help those working with brain organoids to define their choice of model, culture time, functional assay, differentiation, and characterisation strategies.

**Supplementary Information:**

The online version contains supplementary material available at 10.1186/s13287-023-03302-x.

## Background

Human brain development starts in the third week post-conception and continues until early adulthood. Early human brain development progresses through several stages, including the formation of the neural tube (neurulation), the formation of the brain vesicles (ventral induction), and the organisation and structuring of different brain regions. Much of our knowledge on human brain development has been extrapolated from animal studies, mainly drosophila and rodents [1, 2]. Although some developmental features and principles are evolutionarily conserved across species, many features are species specific, including the presence of specific cell populations or broad morphological features. For instance, outer radial glia (oRG), a population of basal unipolar precursor cells [3] which is directly related to the multiple waves of cortical neurogenesis, is only present in higher primates [4, 5]. Another noteworthy difference is the gyrification of the brain in higher primates but is absent in rodents [6]. Expansion of the cortical surface through the formation of gyri and sulci is observed in several different mammal species [7, 8], but is strongest in higher primates and particularly in humans [9]. As a result of these crucial differences, most animal models frequently fail at translating human pathology.

**Fig. 1:**
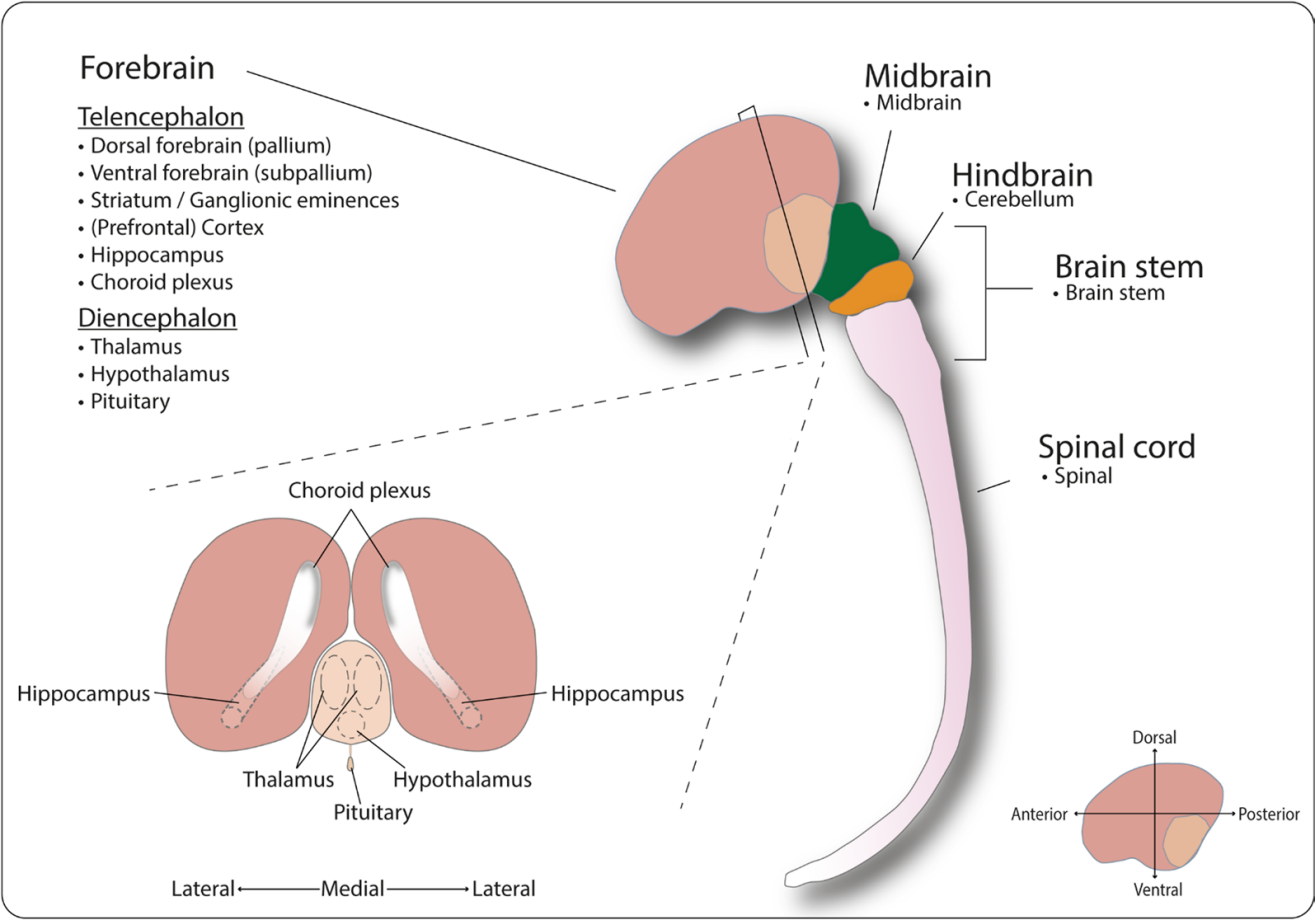
Schematic overview of the currently available brain organoid models representing different regions of the human developing central nervous system. The CNS is represented by the forebrain (in dark and light brown), midbrain (green), hindbrain (orange), and spinal Cord (pink). Below each region, the available organoid models are listed with bullet points. Forebrain organoid protocols are subcategorised under telencephalon (dark brown) and diencephalon (light brown) based on the origins of their respective structures. In the forebrain coronary section, the hippocampus is bilaterally depicted with dashed lines in the telencephalon hemispheres. Lining the ventricles is the choroid plexus epithelium (grey line). In the diencephalon, thalamus and hypothalamus are indicated by dashed lines

Until the last decade, available models to study the human brain development included post-mortem material at different stages of development, extrapolations from animal models [1, 10, 11], and in vitro mono- or co-culture models of cell types present in the brain [12], each presenting their own advantages and limitations (Table 1). In the past decade, the quest for more complex and physiologically relevant human in vitro models for disease modelling and drug discovery [13] culminated in the development of brain organoids (Fig. 1). Considering the characteristic human differences, this review will solely discuss human brain organoids.

**Table 1:** Advantages and limitations of post-mortem material, animal models, and mono- and co-culture cells models to study human brain development

	Advantages	Limitations
Post-mortem material	Human origin	Fixed temporal representation
	True clinical representation	Material can be scarce
Animal models	Allows for over-time studies	Non-human representation
	Systematic model	Requires genetic modifications
	Scalable	Carry ethical burdens
Mono- and co-culture models	Human origin	Usually two-dimensional with limited three-dimensional capabilities
	Scalable	No complex culture conditions
	Long-term sampling

Human brain organoids are self-assembled three-dimensional (3D) tissue models derived from pluripotent stem cells (PSC) that recapitulate certain aspects of human brain development and physiology [14–16], including specific cell types and brain regions. As such, cells can communicate with other cell types and with the extracellular matrix creating a physiological microenvironment [17–19]. Their gene expression profiles resemble that of the human foetal brain, up to the last trimester of gestation [20, 21]. Additionally, they can provide insights into the migratory trajectories of certain cell types in vivo, for example, the migration of interneurons from the ventral forebrain into the dorsal forebrain [22]. Brain organoids have been shown to be a suitable model for studying human neurodevelopment and have been widely used to answer biological questions. They have allowed researchers to gain better understanding of multiple topics, such as the genetic mechanisms driving human brain evolution [23–26], the effect of pollutants on brain development [27–29], and of the neurotrophic effects of multiple drugs [30–32]. Additionally, human brain organoids have helped to understand the neurological impact that a variety of viruses can have on the brain (reviewed by Depla et al. [33]), including the recent SARS-CoV-2 virus [34–36].

Brain organoids can be obtained using guided or non-guided approaches [37–39]. In both approaches, hPSCs are first cultured in 3D spheres called embryoid bodies (EB) which have the ability to differentiate into the three embryonic germ layers: endoderm, mesoderm, and ectoderm. EB are guided towards an ectodermal fate and further differentiated into neural ectoderm which gives rise to neural precursor cells (NPC, neural stem cells and neural progenitors). NPC further differentiate into the diverse neuronal and glial cell types (e.g. neurons, astrocytes, and oligodendrocytes) over the span of the organoid maturation while these cell types organise into region-specific structures mimicking different regions in the human brain. Given their ectodermal origin, organoids generally lack non-ectodermal cell types such as microglia [40] and vasculature [41, 42]. Non-guided protocols typically rely solely on self-organisation and cell-to-cell interactions to generate cerebral organoids. Cerebral organoids mainly display a dorsal forebrain identity, but can also contain cells from other brain regions, such as hippocampus or retina [37]. Guided approaches make use of patterning factors to mimic in vivo development and generate region-specific brain organoids. Generally, these protocols make use of dual Suppressor of Mothers against Decapentaplegic (SMAD) inhibition (bone morphogenetic protein (BMP) and the transforming growth factor beta (TGFβ) pathways) to generate neural ectoderm and then further guide the EB towards the desired identity [43]. During brain development, the anterior–posterior orientation is established by high concentrations of wingless/integrated (WNT) at the posterior side and by anterior inhibition of Wnt signalling by secreted frizzled-related protein 1 (Sfrp1). The dorsal–ventral axis is determined by a high BMP concentration dorsally and a high sonic hedgehog (SHH) concentration ventrally. Similarly, to generate dorsal forebrain organoids EB are treated with SMAD inhibitors with along with Wnt and Shh inhibitors to achieve the desired dorsal anterior identity. On the contrary, the generation of ventral forebrain organoids relies on SHH agonists and Wnt signalling inhibition to obtain ventral anterior-oriented neural ectoderm.

Since the first reports of cerebral and cortical (dorsal forebrain) organoids by Lancaster et al. [37] and Kadoshima et al. [38], respectively, many protocols have been published that make use of patterning factors to generate region-specific organoids [22, 39, 44–47]. This surge in new models led to the appearance of new terms associated with these models, such as brain organoids, cerebral organoids, cortical spheroids, and cortical organoids. Additionally, due to the broad application of these models, multiple modifications and adaptations have been introduced to the original protocols.

Brain organoids are complex models, and given the number of different models, choosing the appropriate one as well as correctly reporting the obtained results from them can be delicate. Even though many valuable reviews have been published on how brain organoids recapitulate brain development and how they can be applied to a myriad of research topics [48–52], to date there is no systematic review available that focuses on the practical aspects of brain organoid technology. Such an overview, including a categorical report of cell types described in each model and their cellular markers, may be valuable for researchers in the field. Here, we present an overview of the available models and their applications. We further focus on articles studying neurodevelopment using brain organoids to assess their described functional characterisation assays and which assays are mostly used. We also provide a protocol comparison of the major organoid models, quantitatively describe the reported small molecules used for forebrain identity induction, and the cell type compositions reported at different stages of organoid maturation. Lastly, we provide an analysis of what immunofluorescence (IF) markers are used to identity each of the cell types. This review does not focus on comparing the specific culture steps for brain organoid generation and differentiation, described in the included research articles and protocol articles. This review serves as a practical guide to better understand the available brain organoid models at hand for neurodevelopmental studies regarding their culture duration, present cell types, and IF characterisation.

## Methods

### Protocol and search strategy

**Fig. 2:**
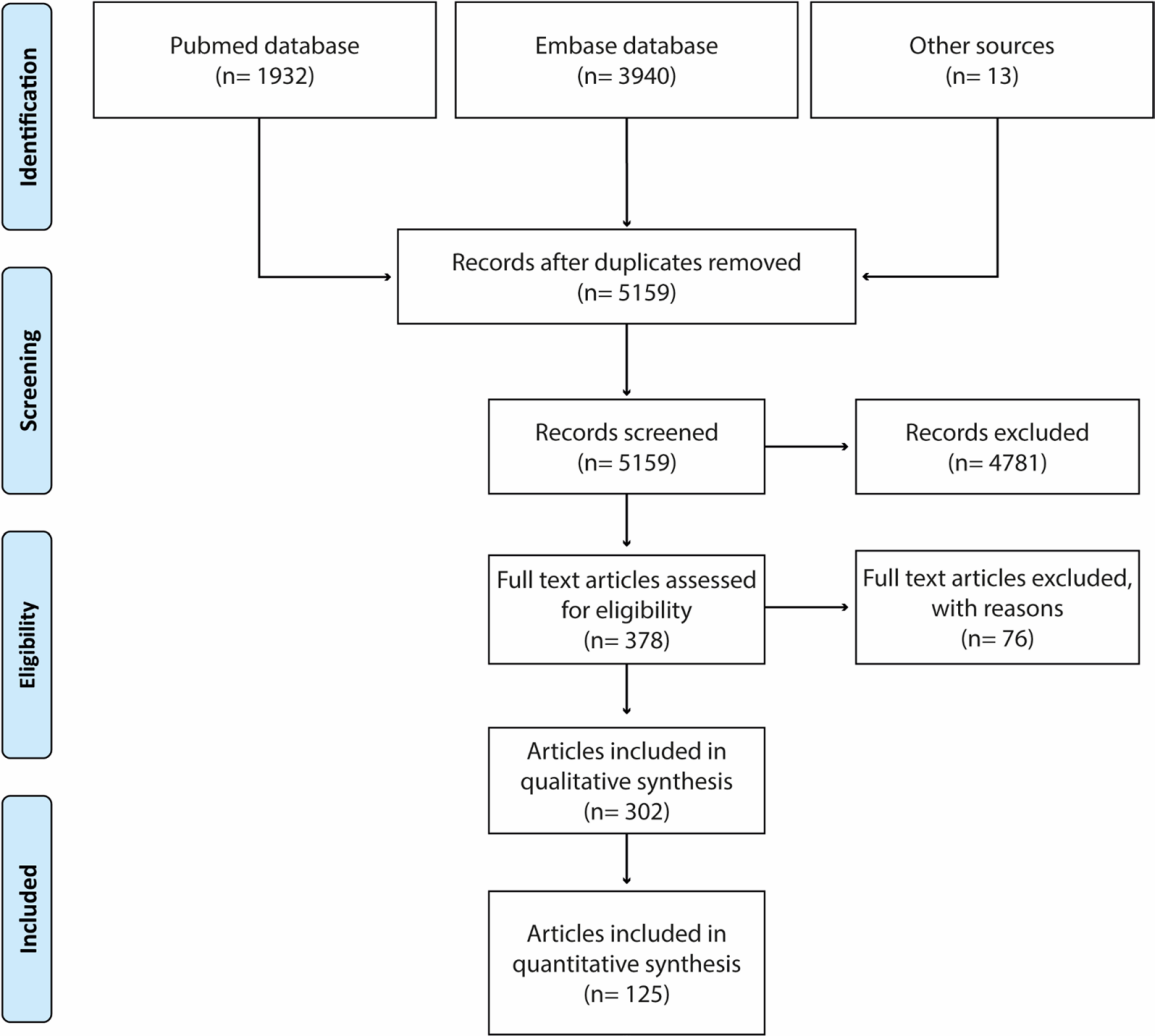
Preferred Reporting Items for Systematic Review and Meta-Analysis for Protocol 2015 article inclusion flow chart

The setup of this systematic review was based on the Preferred Reporting Items for Systematic Review and Meta-Analysis for Protocol 2015 [53] (Fig. 2). PubMed and Ovid Embase (Embase classic and Embase) were used to construct the article base. Articles were obtained from January 1^st^ 2010 up until December 31st 2020 using the search terms described in Table 2. Articles published in 2021, but available in the online databases in 2020 were also included. PubMed and Ovid Embase require different search strategies. For PubMed, the first search (#1) was performed to look for articles associated with the “organoid” mesh term, and synonyms found in the title (ti) or abstract (ab) section. The second search (#2) was performed to search for articles associated with the “Brain” mesh term and brain-related identities in title or abstract. Then, (#3) using the Boolean Operator AND, articles containing both these terms were filtered. For Ovid Embase, the first search established the articles listed under “Organoids”; the second search determined the articles with all valuable keywords present in the title, abstract, or keywords (kw) section. Then, using the Boolean Operator OR, articles obtained through either search were collected. Search results were filtered for peer-reviewed and English-written articles before exportation to Rayyan QCRI Review tool [54]. Duplicates were removed by the program upon importing. A few articles were later included as these were not captured in either search but were obtained through cross referencing. The systematic review was not pre-registered.

**Table 2:** Search terms used to obtain the articles used from the PubMed and Ovid Embase databases

Search	PubMed	Ovid Embase
#1	"Organoids"[Mesh] OR organoid* [tiab] OR spheroid* [tiab] OR self-organised [tiab] OR self-organized [tiab]	Organoids/
#2	("Brain"[Mesh] OR brain* [tiab] OR cortica* [tiab] OR cortex [tiab] OR cerebral* [tiab] OR telencephal* [tiab] OR diencephal* [tiab] OR mesencephal* [tiab] OR rhombencephal* [tiab] OR cerebel* [tiab] OR 'spinal cord' [tiab] OR forebrain [tiab] OR hindbrain [tiab] OR midbrain [tiab])	(Brain organoid* or cerebr* organoid* or cerebel* organoid* or ((forebrain or hindbrain or midbrain or cortical* or cerebel* or cerebr*) adj organoid*) or ((brain or cerebr* or cerebel* or cortical*) adj spher*) or ((telencephal* or diencephal* or rhombencephal* or mesencephal*) adj organoid*) or ((self?organi?* or "self organi?*" or guided) adj2 organoid*) or ((self?organi?* or "self organi?*") adj2 tissue)).ti,ab,kw

### Selection criteria

Articles were included based on the following criteria: articles on brain organoids generated from (induced) hPSC through a fully 3D EB differentiation protocols; original articles and protocol articles; articles using in-house generated organoids. Articles in the following categories were excluded: articles describing organoids with in-between two-dimensional differentiation steps; articles using organoids differentiated from primary or cancer cell lines; reviews, commentary, news articles, and conference articles; in silico studies and articles receiving organoids from other groups or solely describing other datasets; reports describing cell aggregates originating from one cell type (e.g. NPCs or neurons), and articles describing the generation of retinal or inner ear organoid models. If an article could be excluded on the basis of multiple criteria, one criterion was chosen since they did not follow hierarchical rules. Two authors (LM and JD) independently assessed the eligibility of each article according to the inclusion and exclusion criteria. Articles were screened on title and abstract and subsequently on full text using the Rayyan QCRI Review tool. Conflicts in inclusion were discussed and resolved through consensus.

### Data extraction

The following data were extracted: author, year of publication, field of application, stem cell type, what protocol was used for organoid generation, brain organoid identity, age of the brain organoid, functional characterisation, used small molecules and growth factors, reported cell types, the time points reported for each cell type, and the reported IF markers for each cell type. Certain articles described the generation and/or application of multiple brain organoid models. In these cases, data were extracted for each organoid model separately.

A quantitative study was performed on full-text articles to categorise the included articles based on the organoid application (Additional file 1). Articles could be eligible for more than one category, and in these instances the defining factor for categorisation was determined by the aim of the study.

Within the ‘Protocol development & Neurodevelopmental studies’ category, articles were quantitatively analysed for organoid model identity, culture duration, functional assays, small molecule use to guide identity, cell type composition of the models, and the IF markers used to characterise each cell type.

Analysis of organoid model identity was based on reported terminology of the organoid by the authors and by the protocols used to generate the organoid. This analysis allowed for grouping of the articles per organoid identity, on which successive analysis was performed. Organoids were categorised as ‘cerebral organoids’ based on author nomenclature in combination with the use of embedment in extracellular matrix (ECM). This was irrespective of the use of small molecules. Similarly, organoids were categorised as ‘dorsal forebrain organoids’, ‘midbrain’, ‘thalamus’, etc., based on terminology used by the authors. ECM embedment or administration was not taken into consideration into this grouping.

A timeline was constructed to present the development in the generation of different region-specific organoid protocols. This timeline was constructed using only the first-time publications of different models.

Articles grouped by organoid identity were further extracted for the culture duration of the organoids identities (ages in days), and for functional characterisation assessment. For culture duration assessment, the latest reported culture time points were extracted per organoid model. Median culture durations per model were then determined.

For functional characterisation assessment, articles that performed either calcium imaging, whole cell patch clamp, or measured extracellular field potentials were analysed. Assays performed on dissociated organoids and cells outgrown from plated organoids were excluded, as these assays no longer obtained information from 3D tissues. For each included article, the type of functional assay and the respective method of sample preparation (whole mount, sections, dissociated organoids, or plated organoids) were determined. Then, the age range of organoids used for each assay was determined per model. For some articles and models, functional data were available in the context of assembloids; in these cases the age was determined by the sum of organoid at the time of fusion plus the time of assembloid culture. Although the following analyses on the models included in this review were only performed on separate organoid models, assembloid data were considered for qualitative assessment of the functional assays specifically.

Next, article analyses on organoid culture protocols, small molecule and growth factor use for identity guiding, cell type compositions, and reported markers were performed on organoid models demonstrating a telencephalon forebrain identity. These models had to be described in more than four articles. The thalamic & pituitary, midbrain, cerebellum, brain stem, and spinal cord organoid models were not included in the analyses as these brain structures do not originate from the telencephalon. The medial pallial/hippocampal organoid model was assigned to dorsal forebrain organoids, and striatal organoid, and GE organoid models were assigned to ventral forebrain (subpallium) organoids. These brain structures originate from the telencephalon during human neurodevelopment.

To summarise the approaches taken for the generation of cerebral, dorsal, and ventral forebrain organoids, protocols were analysed for initial cell seeding density, timing of EB formation, the use of small molecules and ECM, and the usage of static versus rotational culture conditions. Articles describing the generation of EBs as single cells in suspension (e.g. in a flask or plate) without a definite number of cells per EB were not included in the EB seeding density analysis. For this analysis, EB formation and neural induction were considered as two separate steps. When both steps were performed simultaneously (e.g. small molecule administration at the time of single cell seeding), it was classified as neural induction without an EB formation phase.

Cerebral, dorsal forebrain organoid, and ventral forebrain organoid articles were analysed for the use of small molecules and growth factors to guide the organoid models to their respective identities. The individual molecules were scored by the number of articles describing their use. Certain articles described the use of the same small molecule(s) and/or growth factor(s) in multiple culture steps. If the steps were performed with the same intention (e.g. induction of the EB involving multiple steps using dorsomorphin), their use was scored once, as the molecule served the same purpose. If the culture steps involved different aims (e.g. FGF2 for both EB induction and neural tissue proliferation), the use of that molecule was scored separately, since the purpose of application was different. No analysis was performed on cerebral organoid articles as generation of these organoids does not involve the use of identity-guiding molecules.

Cerebral, dorsal forebrain, and ventral forebrain organoid articles were then analysed for reported cell types and their first reported time points in days. In order to examine cell type compositions and reporting times described in different models, articles were further grouped by identity. Cell types were only included when the authors also stated their respective reporting time points. The resulting list of cell types was used for analysis of cell type composition and IF marker characterisation. For IF marker characterisation analysis, articles were extracted per cell type and no longer by model. In order to improve readability of the cell type composition table and the IF characterisation figure, cell types were grouped where possible. Grouping was performed by the nomenclature used by the authors of the articles and/or by their marker expressions (when IF marker profiles overlapped fully). The resulting groups were as follows: ‘Precursors’ include all precursor cells that were mentioned as such by the original authors without further specification. ‘Neural precursor cells’ include neural precursor cells (NPCs), neuro-epithelial cells, and neural stem cells [55]. ‘Radial glia cells’ include both radial glial (RG) cells and apical progenitors (identical markers: PAX6, SOX2, GFAP). ‘Intermediate progenitor cells’ contained both intermediate progenitor cells and basal progenitors if the latter were specified as “basal intermediate progenitors” [56]. Basal progenitors specified to localise in the oSVZ were assigned to the group ‘oRG’. Glutamatergic neurons and GABAergic neurons reported in the models were grouped together with neurons described as excitatory and inhibitory, respectively. Articles that reported on cell types without clarifying what IF markers were used were excluded from IF analysis. Marker characterisation was performed by cumulative scoring of the articles describing the same marker in relation to the same cell type. However, if certain markers were only reported on once to characterise a specific cell type, these markers were removed. If this left the cell type without any markers to characterise it, it was removed from the list as a whole (including the list for cell type composition and time points) to improve cohesion.

## Results

### Database search results and categorisation

Search of PubMed and Embase databases generated a total of 5160 article entries after removal of duplicates. After exclusion based on title and abstract, 378 articles remained and were assessed on a full-text base. Full-text analysis resulted in further exclusion of 76 articles. The resulting 302 articles were included for qualitative analysis (Fig. 2; Additional file 1).

**Fig. 3:**
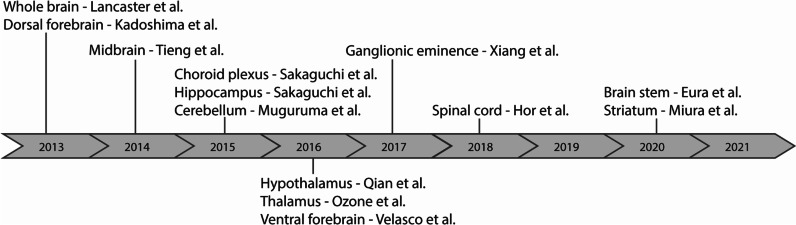
Timeline of the first published protocol of different organoid identities. Only first published articles of each brain organoid identity are presented

Since the initial development of the cerebral and forebrain organoids in 2013, other protocols rapidly followed (Fig. 3). The first following years (2014–2017) a focus on protocols describing the generation of novel dorsal and ventral forebrain models could be observed, including brain regions such as the choroid plexus, hippocampus, and ganglionic eminence (GE). There was an additional focus on midbrain [57] protocols and diencephalon-derived identities like the hypothalamus, thalamus, and pituitary gland. In more recent years (2018–present), novel organoid models were published exhibiting non-forebrain identities, such as the spinal cord [58] and brain stem. Besides the first publications for each model described here, other unique protocols on previously reported regions have been published. One example is the publication on cortical spheroids by Paşca et al. [39] demonstrating dorsal forebrain identity and is a widely used protocol in the field of brain organoid development.

Next, we categorised the articles for better understanding of the applications for which organoids are currently used (Supplementary Table 1). The resulting 302 articles were assigned to one of twelve categories (Additional file 2). Most articles (125/302; 41.4%) were categorised as ‘Protocol development and Neurodevelopment’ studies (from here on ‘Neurodevelopmental studies’). These articles are either the first publication describing a protocol for a certain of brain region organoid or articles that use brain organoids to study human neurodevelopment. The category ‘Protocol optimisation’ (48/302; 15.9%) included articles describing optimisations to brain organoids protocols or use brain organoids to optimise experimental conditions [59–61]. The category ‘Immunology & Infection’ studies (44/302; 14.6%) included articles in which organoids were used for infection studies and/or the subsequent immunological response. The last categories with five or fewer articles assigned included ‘vascularisation’ studies (5/302, > 2%), ‘transplantation’ studies (4/302; > 2%), ‘gene therapy’ studies (2/302; > 1%), and ‘axonal regeneration’ studies (1/302; > 1%). Further quantitative analysis was performed on the 125 articles categorised under ‘Neurodevelopmental studies’ as this category included most studies, as well as publications describing new protocols which generally contain extensive characterisation.

### Neurodevelopmental models and culture duration

**Fig. 4:**
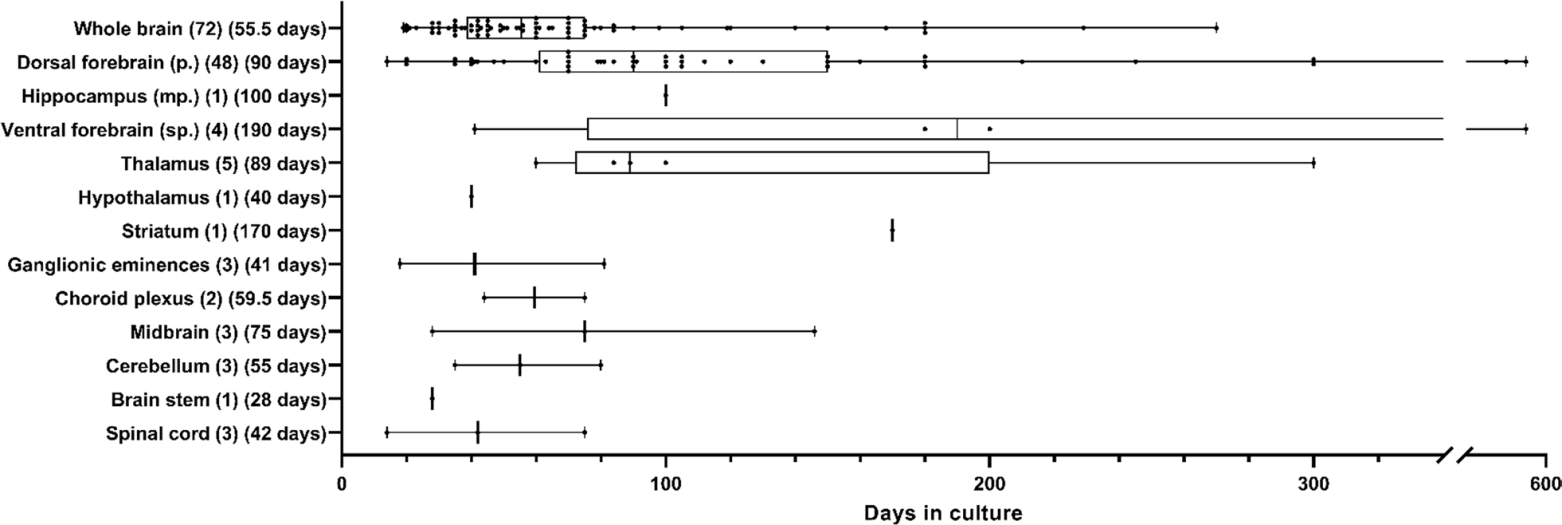
Brain organoid models and their reported days in culture within the neurodevelopmental category are depicted. Box plots depict the 25% and 75% of the individual reports of days in culture, per organoid model. Each report is plotted as a single point. The median days in culture is depicted behind each model for readability. p: pallium, mp: medial pallium, sp: subpallium. The number within brackets depicts the n of articles included per model

To further understand the type of models being used within the ‘Neurodevelopmental studies’ category, we quantified the number of studies using each organoid model (Additional file 2). The most prominent models were cerebral organoids (n = 72), mostly based on the protocol by Lancaster et al. [37, 62], followed by dorsal forebrain organoids (n = 48), based on Paşca et al. [39], thalamic organoids (n = 5), and ventral forebrain organoids (n = 4) (Fig. 4). Certain brain organoid models were only reported once, namely brain stem [63], striatum [47], medial pallium/hippocampus [17], and hypothalamic organoids [64]. One brain organoid protocol was not included for further analyses because no clear identity was reported by the authors [65].

Organoids can be cultured up to several months or even years, which impacts their cellular composition and maturation stage. To determine the culture durations mostly used in the literature, we extracted the latest time point in culture reported for the articles of each model (Fig. 4).

Several articles report on the long-term culture of organoids, with the following maximum times reported for different organoid models: cerebral organoids: 270 days [66], dorsal forebrain organoids: 595 days [67], medial pallium/hippocampal organoids: 100 days [17], ventral forebrain organoids: 595 days [67], thalamus: 300 days [68], hypothalamus: 40 days [64], striatum: 170 days [47], GE: 81 days [69], choroid plexus: 75 days [70], midbrain 146 days [18], cerebellum: 80 days [71], brain stem: 28 days [63], spinal cord: 75 days [46]. To understand the effects of long-term culture on the organoids, we analysed articles that cultured them over 100 days. For cerebral organoids, dorsal forebrain, and ventral forebrain organoids, and thalamic organoids, further maturation of the organoids resulted in increased complexity of cell type compositions and layer formations [46, 66, 67, 72–76] as compared to earlier time points. Extensive astrogenesis (appearing around 60 days, and intensifying after 100 days, of culture) is prominently mentioned for cerebral organoids and dorsal forebrain organoids [39, 69, 77–80]. The appearance of oligodendrocytes and their subsequent myelination of neurons was described to start between 60 and 100 days of culture [72, 73, 79, 81]. A good example of the relationship between maturation and function is illustrated in thalamic-pituitary organoids where, after 100 days, more hormone-producing cells were observed but only became hormonally active around 200 days [68]. Similarly, long-term culture of cerebral organoids (270 days) leads to the presence of electrophysiologically active neurons exhibiting functional synapses and dendritic spines [66]. Astrocytes from dorsal forebrain organoids were shown to express more mature gene expression profiles after 299 + days [79], and dorsal forebrain organoids were reported to demonstrate electrophysiological profiles with nested oscillations after 180 days [74].

### Analysis of neuronal function in brain organoids

Neuronal activity is a key determinant of function in brain organoids. To further understand how this aspect has been analysed in organoid studies, we examined the number of articles describing analyses of neuronal activity using whole cell patch clamp, calcium imaging, or extracellular activity measurements. Overall, we found that only 32 of the 124 articles described the performance of activity assays (Additional file 2). Calcium imaging and patch clamp were the most frequently used assays (in 19 articles each), whereas measurements of extracellular activity were only performed in 6 articles (Additional file 3). To analyse which organoid preparation method was used for each assay, we cross-referenced the method of organoid preparation with the essay performed (Additional file 3). Our analysis shows that whole mount organoids are the most used approach for calcium imaging (11/19) and extracellular activity (4/6), whereas patch clamp is mainly performed in organoid sections of 250 to 350 micron (11/19). Subsequent analysis focused on studies using whole mount and organoid sections as data collected from these approaches are from cells within the 3D structure. Consequently, this led to the exclusion of some models, including cerebellum, hippocampus, and medial pallium. Lastly, we quantified the number of times each assay was used per organoid model and the range of ages at which assays were performed (Table 3). Overall, neuronal activity was shown in all models. In dorsal forebrain organoids, synchronised calcium transients have been described at 45 days up to 175 days in culture. However, one study reported a lack of synchronisation and maturation in day 76 organoids [75]. In cerebral organoids, electrophysiological properties have been detected at 34 days and electrophysiological signature of mature neurons was described at day 62. Calcium imaging data showed the presence of functional cortical networks in day 85 organoids. In contrast, time series studies measuring extracellular field potentials specifically reported cortical networks to only be present after 120 days in culture, which correlated with increased expression of pre- and post-synaptic markers, as well as of other genes involved in synaptic maturation. The presence of functional neural networks was described in thalamus (day 49) and ganglionic eminence organoids (day 40–50). Spinal cord assembloids were shown to generate functional neuromuscular junctions and elicit spontaneous and spontaneous activity in muscle cells. Additionally, they were shown to receive input from the dorsal forebrain organoids and form functional circuits. In midbrain organoids, electrophysiological properties characteristic of dopaminergic (DA) neurons have been described and could be inhibited by D2/D3 agonists. Lastly the only report on brain stem organoids reported that at day 30 most cells did not display action or membrane potential and only a few cells were responsive.

**Table 3:** Overview of neuronal activity assays performed per organoid model and respective age range

Organoid model	Functional assay	Total analyses	Age range (days)
Cerebral	Calcium [37, 82]	2	NS
	Patch [83, 84]	2	61–121
	Extracellular [66, 85]	2	34- 244
Dorsal forebrain	Calcium [39, 46, 47, 64, 69, 75, 86, 87]	8	45–109
	Patch [22, 39, 64, 81, 86, 88, 89]	7	51–175
	Extracellular [89]	1	165–175
Midbrain	Patch [18]	1	33–84
	Extracellular [57]	1	28
Medial ganglionic eminence	Calcium [69]	1	40–50
	Patch [69]	1	NS
Spinal cord	Calcium [46]	1	69–96*
	Patch [46]	1	45–75
Thalamus	Calcium [90]	1	49
	Patch [90]	1	90–100
Striatum	Calcium [47]	1	90–135*
	Patch [47]	1	110–170
Brain stem	Patch [63]	1	92
Ventral forebrain	Calcium [22, 87]	2	43–52
	Patch [22]	1	125–194*

^*^Range includes data from assembloid studies and was determined by the sum of organoid age at time of fusion plus the time of assembloid culture before assay

*NS* not stated

### Cerebral, dorsal forebrain, and ventral forebrain organoids culture protocols

Next, we focussed on the articles describing cerebral, dorsal forebrain, and ventral forebrain organoids, as these were the most used models. The hippocampal organoid model was assigned to dorsal forebrain organoids, and striatal organoid and GE organoid models were assigned to ventral forebrain organoids, as these brain structures originate from the telencephalon during human neurodevelopment.

To compare different protocols available to generate the cerebral, dorsal, and ventral forebrain organoids, we analysed and summarised key aspects of the protocols, including cell seeding density, neural induction duration, use of small molecules, use of ECM, and presence of rotational culture (Table 4). Cerebral organoids were generally generated as described by Lancaster and colleagues [37, 62], without the use of small molecules and making use of embedment in ECM (Matrigel or Geltrex) and rotational culture systems (orbital shaker or spinning flasks). Some articles, based on protocols by Kadoshima et al. [38], Qian et al. [91], and Coulter et al. [92], described the generation of cerebral organoids with the use of dorsalising or ventralising small molecules. A subset of protocols did not describe the use of ECM or rotation conditions. Protocols for generation of dorsal and ventral forebrain organoid used small molecules to achieve regionalisation of the models. Most of the dorsal forebrain organoids were created without the addition of a supporting ECM; others were either embedded or received liquid ECM added into the culture medium (Matrigel or Geltrex, 0.5–2%). Additionally, a subset of these articles reports the mechanical removal of the ECM several days after embedding, by either cutting off the ECM or pipetting the organoid up and down. None of the ventral forebrain organoid protocols reported ECM use. Dorsal forebrain and ventral forebrain organoid were primarily cultured under static conditions, compared to the majority of the cerebral organoids which were cultured under rotation conditions. Rotation culture was strongly linked to the type of ECM administration, with all the protocols describing embedment of the organoids also describing rotation culture conditions. The EB starting cell number did not seem to influence this choice in culture, which is interesting as this is a more defining factor regarding nutrient diffusion. For all models, we found there to be a large range of initial cell seeding density per EB and therefore of EB starting size and differences in cell seeding densities between protocols.

**Table 4:** Summary table comparing the cerebral, dorsal forebrain, and ventral forebrain organoid culture protocols

	Cerebral organoids	Dorsal forebrain organoids	Ventral forebrain organoids
EB formation	500–90,000 cells/ EB	3000–20,000/ EB	5000–10,000/ EB
	1–7 days	1–10 days	1–10 days
Neural induction	Small molecules	Small molecules	Small molecules
	Yes (16%), No (84%)	Yes (100%), No (0%)	Yes (100%), No (0%)
	2–18 days	4–26 days	5–24 days
Extracellular Matrix	Embedment (93%)	Embedment (8%)	Embedment (0%)
	Liquid ECM (0%)	Liquid ECM (26%)	Liquid ECM (0%)
	No ECM (7%)	No ECM (66%)	No ECM (100%)
Culture conditions	Static (7%),	Static (68%),	Static (50%),
	Rotation (93%)	Rotation (32%)	Rotation (50%)

Key aspects of organoid generation are compared between different models

### Small molecule use to guide brain region identities

**Fig. 5:**
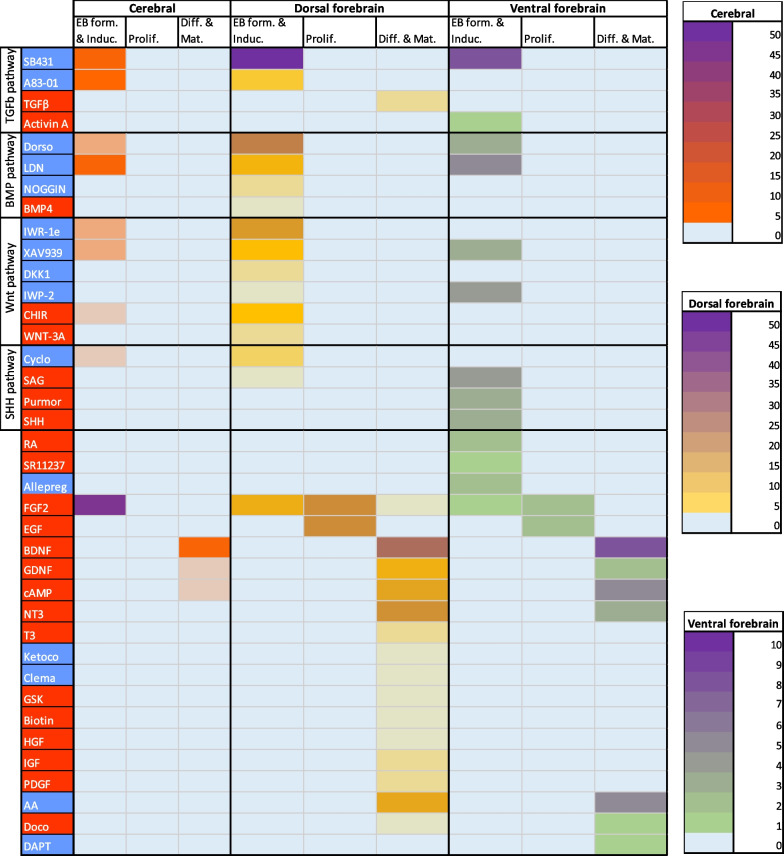
Small molecule and growth factor used in guided dorsal forebrain and ventral forebrain protocols. Molecules and factors are scored by their uses in EB formation and induction of neuroectoderm, proliferation of the neural tissues, or differentiation and maturation of the organoids. Molecules are grouped by their pathways and determined to exert an inhibitory (blue) or stimulating (red) effect on different pathways. Abbreviations top to bottom, left to right, form: formation, Induc: induction, Prolif: proliferation, Diff: differentiation, Mat: maturation, SB431: SB-431542/3, Activin A: Recominbant Human/ Mouse/ Rat Activin A, Dorso: dorsomorphine, LDN: LDN-193189, IWR-1e: IWR-1(endo), CHIR: CHIR99021, Cyclo: cyclopamine, SAG: smoothened agonist, Purmor: purmorphamine, SHH: Recombinant SHH, RA: retinoic acid, Allepreg: allepregnanolone, Ketoco: ketoconazole, Clema: clemastine, GSK: GSK2656157, HGF: hepatocyte growth factor, IGF: Insulin-like growth factor, PDGF: PDGF-AA, AA: ascorbic acid, Doco: docosahexaenoic acid

To elaborate on the guiding principles used to generate dorsal and ventral forebrain identities, we analysed and scored different small molecules and growth factors used in each publication (Fig. 5). During data extraction, different stages of organoid generation became apparent. The first stage described the generation of the EBs and their subsequent neuroectoderm induction, followed by an optional proliferation step, and lastly differentiation and maturation. In most dorsal forebrain protocols, dual SMAD inhibition is restricted to EB formation and induction (21/50), since continuous BMP inhibition blocks dorsalisation of the tissue [93]. Some articles described the additional use of Wnt inhibitors (11/50) alone, or combined with Shh activators (2/50). One article described the timed use of Wnt inhibitors and activators to specifically generate medial pallium/ hippocampal tissue [17]. Single SMAD inhibition was also mentioned in combination with Wnt inhibition (14/50) or SHH inhibition (2/50). For all cases of single SMAD inhibition, the TGFβ pathway was inhibited.

Dual SMAD inhibition was also described for ventral forebrain organoid induction, with the additional administration of Wnt inhibitors (1/8) alone, or with Wnt inhibitors and Shh activators combined (8/8). Retinoic acid receptor is highly expressed in the striatum during brain development [94]. Two of the nine articles describing dual SMAD inhibition together with Wnt inhibition and Shh activation described the use of RA signalling activation with RA for differentiation of subpallial organoids [22, 67], and one article described the use of the RA receptor agonist SR11237 for striatal organoid [47].

The use of molecules for neural tissue proliferation was similar for dorsal forebrain organoids (FGF2 (21/50) and epidermal growth factor (EGF) (21/50)), and ventral forebrain organoids (FGF2 (2/8) and EGF (2/8)). Once identity was achieved, dorsal and ventral forebrain organoid medium was supplemented with growth factors to support neuronal differentiation and maturation. These included brain-derived neurotrophic factor (BDNF), glial cell-derived neurotrophic factor (GDNF), cyclic AMP (cAMP), neurotrophin 3 (NT3), and ascorbic acid (AA). Also, the notch signalling pathway/ gamma-secretase inhibitor DAPT (1/8) was used for differentiation and maturation of ventral forebrain organoids.

To generate oligodendrocyte containing dorsal forebrain organoids, the additional use thyroid hormone 3 (T3) (2/50), insulin-like growth factor (IGF) (2/50), platelet-derived growth factor AA (PDGF-AA) (2/50), biotin (1/50), hepatocyte growth factor (HGF) (1/50), cytochrome-P450 inhibitor ketoconazole (1/50), EBP inhibitor clemastine (1/50), and PERK-inhibitor GSK2656157 (1/50) were described during differentiation and maturation [73, 81]. Small molecules ketoconaloze, clemastine, and GSK2656157, along with T3 and PDGF-AA, were used to stimulate myelination and oligodendrocyte maturation [73].

A few publications on cerebral organoids (12/72) reported the use of patterning factors. Of these, most reported the use of dual SMAD inhibition alone (5/12), whereas some used it in combination with Wnt inhibitors (3/12) or Wnt and Shh inhibitors (1/12). Single SMAD inhibition, targeting the TGFβ pathway, in combination with Wnt inhibition was described once. One article reported on the use of Wnt inhibitors only without SMAD inhibition and one article on Shh inhibition only without SMAD inhibition. Lastly, one article reported ventralisation using only Wnt inhibitors and Shh activators, without SMAD inhibition. Regarding cerebral organoid maturation, some protocols described the (continued) use of TGFβ (1/72), IWR-1e (1/72), cyclopamine (1/72), BDNF (7/72), GDNF (2/72), cAMP (2/72), and AA (1/72). One article described the use of an SHH-producing regionaliser made from modified iPSCs [19] to induce a ventral identity.

Next, we categorised whether the molecules used were of synthetic or natural origins. Overall, for SMAD inhibition, the predominant choice to block TGFβ signalling pathway described in all three models was by using synthetic molecules SB431542/3 (65/68 total articles) or A83-01 (10/68 total articles). In contrast, the choice of BMP inhibitors differed between cerebral, dorsal, and ventral protocols. In dorsal forebrain protocols, dorsomorphin was the preferred choice (25/36), followed by synthetic chemical LDN-193189 (9/36) or NOGGIN (2/36). Contrarily, in ventral and cerebral organoid protocols, LDN-193189 (5/8 and 7/10, respectively) was preferred over dorsomorphin (3/8 and 3/10, respectively). Activation of BMP pathway was only described in one dorsal forebrain article using BMP4. The use of Wnt inhibitors was also different across organoid identities, with dorsal forebrain protocols describing the use of synthetic inhibitors IWR-1(endo) (17/26), XAV939 (6/26), and IWP-2 (1/26), or natural inhibitor DKK1 (2/26). Similarly, cerebral organoid protocols solely mentioned the use of synthetic inhibitors IWR-1e (3/7), XAV939 (3/7), and IWP-2 (1/7). In contrast, protocols for ventral identities reported on the use of XAV939 (3/7) and IWP-2 (4/7) only. Wnt activation was only described in cerebral and dorsal forebrain organoids and was achieved using the synthetic molecule CHIR99021 (2/3 and 5/7, respectively) and naturally occurring WNT-3A (1/3 and 2/7, respectively). Lastly, Shh inhibition for dorsalisation was exclusively done using cyclopamine in cerebral and dorsal forebrain organoid models (2/2 and 3/3, respectively). One cerebral organoid and one dorsal forebrain article described smoothened agonist (SAG) treatment for Shh activation. For ventral forebrain organoids, Shh activation was primarily achieved using SAG (4/8), followed by recombinant SHH (3/8) or synthetic inhibitor purmorphamine (3/8).

A point worth noting is that we noticed apparent differences in concentrations of the same molecule, when analysing the dorsal forebrain and ventral forebrain organoid protocols. We also noticed apparent differences in the number of days that the molecule was administered to obtain similar effects with regard to pathway inhibition or activation.

### Categorisation of present cell types and their reported time points

We then set out to investigate the cellular composition of different forebrain organoid models. Articles grouped by identity were extracted for the reported cell types and their cognate first reporting times (Table 5). As the dorsal forebrain is the most prominent brain region present in cerebral organoids, a large overlap was observed between them and dorsal forebrain models when looking at neuronal subtypes. Given their unguided nature, cerebral organoids have also been described to contain ventral forebrain, midbrain, and choroid plexus (cuboidal epithelium) identities, as well as microglia. Dorsal and ventral forebrain organoids contain only region-specific cell types. One exception is the report of MGE precursors in dorsal forebrain organoids [69]. Neural precursor cells were reported around similar time points on cerebral and dorsal forebrain organoids [30 and 35 median days, respectively], whereas this was earlier in ventral forebrain organoids (median 20 days). Neurons were reported in cerebral organoids as early as seven days of culture [95], and mature neurons were reported at 28 days. In dorsal forebrain organoids, neurons were reported from 14 days onwards, although one article described the presence of mature neurons after twelve days [96]. In ventral forebrain organoids, neurons were reported after 25 days. First reports of astrocytes started at 30, 76, and 80 days in cerebral, dorsal forebrain, and ventral forebrain organoids, respectively. Mature astrocytes were reported in dorsal forebrain organoids starting from 120 days and have not been reported in the other two models. Oligodendrocytes were reported in 28-day-old cerebral organoids and mature oligodendrocytes after 39 days. In dorsal and ventral forebrain organoid, oligodendrocytes have been reported at 98 and 80 days, respectively.

**Table 5:** Organoid models and reported cell types

	Range in reported days per cell type| median day		
Cell types	Cerebral (n = 61)	Dorsal (n = 38)	Ventral (n = 6)
*Progenitors*			
Progenitors	15–85 | 35	12–90 | 35.5	75–85 | 80
Neural progenitor cells	07–70 | 30	12–125 | 35	20–20 | 20
Radial glial cells	15–84 | 32.5	18–245 | 35.5	20–20 | 20
Outer radial glial cells	14–84 | 47.5	20–125 | 60	
Intermediate progenitor cells	19–70 | 35	28–70 | 54	
Cortical progenitors	19–45 | 33	41–41 | 41	
Hypothalamic precursors	70–70 | 70		
GE progenitors			
MGE precursors		18–18 | 18	18–18 | 18
Dorsal forebrain-specific progenitors	14–14 | 14	13–79 | 14	
Dorsal telencephalic progenitors	21–21 | 21	37–37 | 37	
Interneuron precursor			71–71 | 71
Oligodendrocyte progenitors	30–30 | 30	50–51 | 50.5	
*Neurons & glia*			
Neurons	07–180 | 30	14–125 | 40.5	25–80 | 41
Mature neurons	28–112 | 31	12–81 | 58	
Excitatory neurons	19–180 | 63	46–180 | 90	
Inhibitory neurons	19–180 | 35	35–180 | 84	46–85 | 60.5
DA neurons	30–30 | 30		
Glial cells	180–180 | 180	54–180 | 100	
Astrocytes	30–112 | 60	76–300 | 98	80–80 | 80
Mature astrocytes		120–200 | 160	
Oligodendrocytes	28–180 | 35	98–115 | 106.5	80–80 | 80
Mature (late stage)	39–180 | 109.5	100–100 | 100	
*Neuronal subtypes*			
Neuroblasts (early pioneer neurons)	15–63 | 39		22–22 | 22
Cortical neurons	21–180 | 30	33–112 | 41	
Cajal-Retzius cells	30–75 | 33	28–70 | 42	
Lower layer (deep layers V-VI)	14–84 | 52	28–140 | 70	
Upper layer (superficial layers II-IV)	19–84 | 63.5	52–140 | 70	
Forebrain neurons	25–25 | 25	65–65 | 65	
Striatal (cortical) interneurons	70–70 | 70		
GE young neurons			
Cortical (pre)plate neurons	60–60 | 60		
*Others*			
Cuboidal epithelium	40–40 | 40		
Microglia	24–39 | 31.5		

Articles describing the use of cerebral organoid, dorsal forebrain organoids, and ventral forebrain organoid models were summarised and analysed for each reported cell type. The range of reported time points per cell type was extracted from articles belonging to either of the model categories. Per cell type, the median day of reportage of that specific cell type is depicted

### Markers used for characterisation of different cell types

**Fig. 6:**
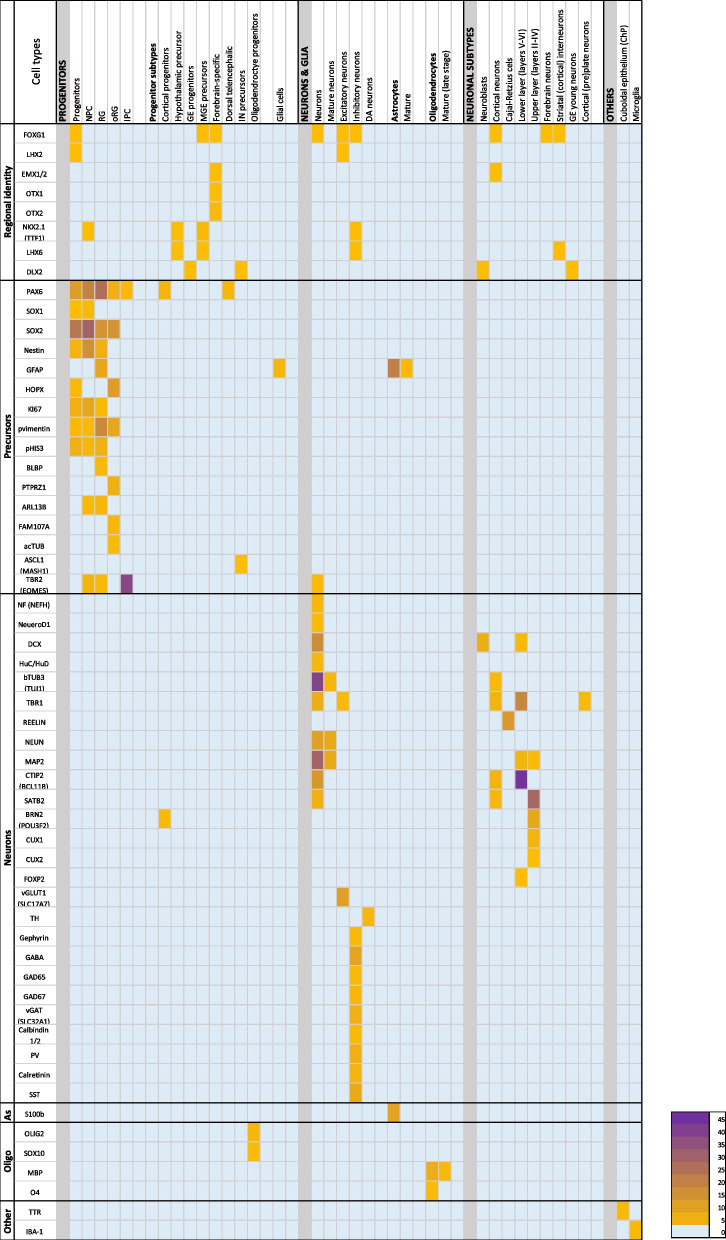
Cell types and their reported markers*.* Individual markers are depicted in relation to the cell type that they were reported to characterise. The ‘Regional identity’ column depicts markers not often used for specific cell type characterisations, but more generally used to determine the identity of the organoid model. Strong marker overlap is evident between precursors cells. NPC: neural precursor cells; RG: radial glia; oRG: outer radial glia; IPC: intermediate progenitor cells; GE: ganglionic eminence; MGE: medial ganglionic eminence; IN: interneuron; DA: dopaminergic; ChP: choroid plexus

To provide an overview of the most used cell markers, we summarised the reported markers used for cell type characterisation (Fig. 6). Markers used to determine organoid identity were grouped under regional identity. FOXG1 was the commonly used marker to visualise forebrain identity, both dorsal and ventral. Dorsal forebrain was specified by staining for PAX6, LHX2 and EMX1&2. Ventral identities were mostly visualised by NKX2.1, LHX6, and DLX2 expression, which also marked the presences of hypothalamic, GE and MGE precursors, as well as GABAergic inhibitory interneurons and their precursors (Fig. 6). OTX1 & OTX2 are broadly expressed during development, both in ectoderm and mesoderm [97]. In the developing brain, they are mostly expressed in the mesencephalon and forebrain, midbrain, and hindbrain. In brain organoids, OTX1 and OTX2 were used for determination of forebrain, dorsal forebrain, ventral forebrain.

When visualising neural precursors (NPC and RG), SOX2 and Nestin were the most used markers. Dorsal forebrain progenitors were mostly visualised with PAX6, GFAP and phosphorylated vimentin (pVimentin), whereas ventral forebrain precursors can be characterised by NKX2.1. oRG, a cortex-specific population localised in the oSVZ, was usually visualised using HOPX. Intermediate progenitor cells (IPCs) were visualised with TBR2. Markers NEUN and bTUB3 were most used to visualise neurons in general, while DCX and NeuroD1 were used to distinguish immature neurons from MAP2-positive mature neurons. Cortical neurons were distinguished using CTIP2 and TBR1 for deep-layer neurons (layers V – VI) and SATB2 and BRN2 for superficial-layer neurons (layers II – IV). Cajal-Retzius cells, which make up the mantle zone and the cortical plate, were shown specifically using REELIN. Ventral interneuron subtypes were visualised and distinguished by their subtype-specific markers parvalbumin (PV), calretinin, calbindin or somatostatin (SST). Glutamatergic excitatory neurons were shown by staining for vGLUT1 (SCL17A7) and GABAergic inhibitory (inter)neurons by GABA, GAD65 & GAD67, vGAT (SCL32A3). For characterisation of astrocytes, markers GFAP and S100b were the most used. Oligodendrocytes were reported using MBP and O4, and their progenitors with OLIG2 and SOX10.

Markers followed a general cell type-specific application, with specific markers for precursors, neurons, astrocytes, and oligodendrocytes. The IF markers used were not exclusive to one cell type but were able to visualise multiple cell types. pVimentin is regularly used to characterise NPC, yet this marker is also expressed in non-neuronal fibroblasts. It is important to keep in mind the aim of the study when using these markers whether it is only necessary to indicate neuronal presence (MAP2, bTUB3, NEUN) or a specific neuronal subtype (e.g. neuroblasts or Cajal-Retzius cells). The overlap in markers was especially evident in precursor cell types; NPCs, RG, neural stem cells, and neuroepithelial cells were all reported by staining against PAX6, SOX2, and Nestin.

## Discussion

This review aimed to systematically summarise brain organoid model applications, usage, and cell composition. We found that most articles using brain organoids fall under neurodevelopmental studies, protocol optimisation studies, or immunology and infection studies. However, brain organoid technology has been used for many other applications, including gene therapy [98, 99], psychiatric disorders [100–108], transplantation studies [109–112], cancer studies [113–122], and brain therapeutic studies [30–32, 123–129]. This illustrates their broad applicability in neuroscience research.

Within the neurodevelopmental field, cerebral and dorsal forebrain organoids are the most used models. As cerebral and dorsal forebrain organoids were the first models to have been reported, researchers may be mainly acquainted with these models. Organoids have been cultured for considerably long periods of time (> 100 days). This long culture duration resulted in brain organoid models that exhibit diverse, mature, and cell–cell interactions (e.g. myelination). These brain organoids exhibited extensive cellular organisation and layer formation with mature gene expression profiles. Nevertheless, even with long culture times, brain organoids display a foetal to early postnatal stage phenotype overall [130, 131]. Transcriptome analysis on brain organoids and primary material demonstrates increased metabolic stress in brain organoids and is proposed to contribute to impaired molecular subtype specification of the individual cell types [132]. Currently, brain organoids are of limited use to model adult neurodevelopment, as opposed to modelling the early gestational period. Nevertheless, brain organoids have been extensively applied to study human neurodegenerative disorders [133–135].

The culture durations and maturation stages of the organoids are intimately related to their cellular compositions. However, in this review we did not analyse this relationship in detail. Relating the cell type occurrence to the median culture durations of the three models, we can conclude that cerebral organoids are generally cultured up to the times when most cell types are present [35–60 days]. Dorsal forebrain organoids have a median culture duration of 84 days at which stage they contain astrocytes and potentially (immature) oligodendrocytes. Ventral forebrain organoids are cultured for rather long periods (median 125 days) at which stage all cell types are present. Even though reports on long-term cultures provide an estimate of how long organoids can be kept in culture, it would be interesting to determine their long-term viability. However, most studies included in our analysis do not state the specific reasons for which cultures are terminated or why culture time is not extended. Therefore, this could not be extracted from our analysis.

Regarding the assessment of neuronal function, studies using different methods report different outcomes on maturation of neuronal networks. As assays and sample preparation methods differ across studies, even within the same model, it is difficult to extrapolate general conclusions. Notably, most studies included in our analysis use functional assays as a proof of principle for the presence of functional neurons. Further in-depth studies using different assays for characterising the electrophysiological properties of organoid models over time would be crucial to precisely determine the emergence and maturation of active, and thus functional, neuronal networks. Nonetheless, we found that neuronal activity is present in all models evaluated and that most studies report maturation of neuronal networks overtime, indicating that organoid maturation is accompanied by increased connectivity.

With regard to the application of small molecules for organoid regionalisation, it would be important to further examine their individual and combined efficacies and standardise their uses. Even though they might be interchangeable with regards to function, molecules with different half-lives and stabilities may exert different influences on seemingly similar protocols, especially taking into consideration that the use of the same molecule is described in different protocols, with different concentrations, and/or over different spans of time. Furthermore, the morphogen pathways manipulated at the initial stages of the organoid differentiation are crucial for determining the regional identity. Changes at this initial stage may lead to different positional identities within the same brain region. This is particularly relevant for dorsal forebrain organoids where some protocols use only dual SMAD inhibition and others use additional dorsalisation and anteriorisation cues, such as Wnt and Shh inhibition. Further studies elaborating on the efficiency and efficacy of different combinations of synthesised and natural molecules, their concentration, and application period would be highly relevant for the standardisation of brain organoid models.

Overall, we provided a summary of the principles and critical steps used to generate cerebral, dorsal, and ventral forebrain organoids. In performing this analysis, we encountered several inconsistencies which may account for overall differences across protocols. For instance, across the three models we observed a large variation regarding the initial EB size (seeding density). Another key determinant of organoid differentiation is the use of ECM support. This varies mostly in cerebral organoids where specimens are either embedded in ECM droplets; ECM is added to the medium or is completely absent. Each of these approaches has advantages and disadvantages, whereas droplet ECM allows for a physical matrix environment for the organoid to expand in. Liquid ECM administration to the medium does not provide an instant 3D matrix environment. It is important to mention that Matrigel- and Geltrex-based ECM have large inter-batch variability which impacts protocol outcomes [136]. As such, approaches lacking ECM support are not subjected to such variability. The use of rotational cultures is advantageous as it increases the diffusion of nutrients and oxygen to the core of the organoid [62, 137, 138]. However, despite this, organoids are still reported to have a necrotic core after they reach a certain size limit [37]. Additionally, the use of this type of culture requires specialised equipment that may limit the use of this technique in some laboratories. In summary, each model and approach have its own advantages and disadvantages (Table 6) that should be considered when choosing the appropriate model.

**Table 6:** Advantages and limitations of the cerebral, dorsal forebrain, and ventral forebrain organoid models

Model	Advantages	Limitations
Cerebral organoids	Extracellular matrix support for neuroectoderm expansion	Spontaneous differentiation of different brain regions
	Presence of multiple brain region characteristics	Primarily dorsal forebrain identity
		Heterogeneous in size
Dorsal forebrain organoids	Specific regional identities	No extracellular matrix embedment
	Homogeneous in size	
	Possibility to fuse with other regional organoids to create more complex systems	
Ventral forebrain organoids	Specific regional identities	In most cases, lack of embedded extracellular matrix support
	Homogeneous in size	
	Possibility to fuse with other regional organoids to create more complex systems	

Considering the reportages of the individual cell types, all cell types should be present after 80 days of culture in cerebral and dorsal forebrain organoids. For ventral forebrain organoids, it is difficult to draw such a conclusion as fewer articles reported on this model. In our final analysis, we examined the IF markers used to characterise each cell type in cerebral, dorsal, and ventral forebrain organoids with the intent of providing an easy-to-use template for researchers to select markers to use. Given the lack of standardisation in terminology used to report cell types, a comparison between articles was in some cases difficult. As an example, several reports refer to “dorsal telencephalon precursors” and “cortical progenitors” using identical IF marker characterisation. As such, it would be helpful to clarify and standardise how to refer to each cell type as well as which markers to use for characterisation.

Although this is the first extensive report on the practical aspects of human brain organoid culture and reportage, this review has some limitations. In addition to the PubMed and Ovid Embase database searches performed, a few articles had to be added via cross-referencing later, indicating a possible underrepresentation of articles in the searches. We focussed on a specific category of studies, lowering the number of included articles possibly limiting the downstream analysis. We did not aim to perform a quality assessment of each protocol regarding their validity or quality, as we think that each protocol can have its own advantages and limitations in the context of their use and application. This review’s aim was to report and summarise what is currently described. Per organoid model, we described the culture ranges in days as well as reported cell types. Our analysis did not allow us to determine important aspects of organoids cultures such as the presence of non-neuronal cell types or the long-term viability of the models. These aspects would be important for a critical assessment of the protocols, but unfortunately, they often go unreported. Lastly, IF was chosen as the reference for characterisation since it is a generally accessible technique used by most laboratories for tissue characterisation. However, not every article included our analysis applied this technique.

Future expansion and optimisation of brain organoid technology can be expected in the coming years to address several outstanding limitations in the field including the integration of vascularisation [42, 139, 140] and additional cell types such as microglia [40, 84, 141]. Additionally, we can expect a continuous focus on tissue organisation to increasingly mimic brain development through the elegant use of assembloids described by Birey et al. [22] and Xiang et al. [69], or the integration of regional organisers as shown by Cederquist and colleagues [19]. Lastly, emphasis on extending the phenotypic state of brain organoids from foetal into more mature phenotypes [131] may also be expected. As the field progresses to address these and other topics, attention should be given to improve intra-model standardisation and standardise the nomenclature used for the cell types.

## Conclusion

The dynamic development of new approaches and optimisation of protocols to generate brain organoids has amounted to a great number of articles and information currently available. In this review, we provide a systematic overview of culture durations, functional activity assays, protocol key aspect comparisons, small molecule and growth factor application, cell type composition, and IF marker usage of the most used models to be used as a practical guide for researchers in the field of human brain organoid research.

## Supplementary Information

**Additional file 1**: Categorisation of included articles. This file contains a table of the 302 articles that were included into the study, and which are subsequently categorised into research fields based on the stated aim of the articles.

**Additional file 2**: Neurodevelopmental and Protocol generation articles. This file contains a table of the 124 articles categorised under ‘Neurodevelopmental studies and Protocol generation’. Each article is analysed for the name(s) of the organoid model(s) reported as well as the identity of each model, the culture durations (age) of each model, and whether the article performed functional assays (Yes/No score).

**Additional file 3**: Functional assays described in different brain organoid models. This file contains a table of the articles included for the functional assay analysis. Articles were analysed for the type of assay described, and for the method of organoid preparation. Articles with an * depict assembloid studies.

**Additional file 4**: The reference list of the articles described in Additional files 1–3.

**Additional file 5**: Individual markers are depicted in relation to the cell type that they were reported to characterise. The ‘Regional identity’ column depicts markers not often used for specific cell type characterisations, but more generally used to determine the identity of the organoid model. Strong marker overlap is evident between precursors cells. NPC: neural precursor cells; RG: radial glia; oRG: outer radial glia; IPC: intermediate progenitor cells; GE: ganglionic eminence; MGE: medial ganglionic eminence; IN: interneuron; DA: dopaminergic; ChP: choroid plexus.

## Data Availability

The datasets used and/or analysed during the current study are available from the corresponding author on reasonable request.

## Abbreviations

3D: Three-dimensional
AA: Ascorbic acid
Ab: Abstract
BDNF: Brain-derived neurotrophic factor
BMP: Bone morphogenetic protein
cAMP: Cyclic AMP
EB: Embryoid body
ECM: Extracellular matrix
EGF: Epidermal growth factor
FGF2: Basic fibroblast growth factor
GDNF: Glial cell-derived neurotrophic factor
GE: Ganglionic eminence
HGF: Hepatocyte growth factor
hPSC: Human-induced pluripotent stem cells
IF: Immunofluorescence
IGF: Insulin-like growth factor
IPC: Intermediate progenitor cells
Kw: Keywords
NPC: Neural precursor cells
NT3: Neurotrophin 3
PDGF-AA: Platelet-derived growth factor AA
PV: Parvalbumin
pVimentin: Phosphorylated vimentin
(o)RG: (Outer) radial glia
RA: Retinoic acid
SAG: Smoothened agonist
Shh/SHH: Sonic hedgehog
SMAD: Suppressor of Mothers against Decapentaplegic
SST: Somatostatin
(o/i)SVZ: (Inner/outer) subventricular zone
T3: Thyroid hormone 3
TGFβ: Transforming growth factor beta
Ti: Title
Wnt/WNT: Wingless/integrated
