# Supplementary material for: A beginner’s guide on the use of brain organoids for neuroscientists: a systematic review
Source: Stem Cell Res Ther. 2023 Apr 15;14:87. doi: 10.1186/s13287-023-03302-x (PMC10105545; doi:10.1186/s13287-023-03302-x)
Supplement: Supplementary file 4 — Additional file 4: The reference list of the articles described in Additional files 1–3. [file 13287_2023_3302_MOESM4_ESM.docx]

Abreu, C. M., Gama, L., Krasemann, S., Chesnut, M., Odwin-Dacosta, S., Hogberg, H. T., . . . Pamies, D. (2018). Microglia Increase Inflammatory Responses in iPSC-Derived Human BrainSpheres. *Frontiers in microbiology, 9*, 2766. doi:10.3389/fmicb.2018.02766

Abud, E. M., Ramirez, R. N., Martinez, E. S., Healy, L. M., Nguyen, C. H. H., Newman, S. A., . . . Blurton-Jones, M. (2017). iPSC-Derived Human Microglia-like Cells to Study Neurological Diseases. *Neuron, 94*(2), 278-293 e279. doi:10.1016/j.neuron.2017.03.042

Akamine, S., Okuzono, S., Yamamoto, H., Setoyama, D., Sagata, N., Ohgidani, M., . . . Ohga, S. (2020). GNAO1 Organizes the Cytoskeletal Remodeling and Firing of Developing Neurons. *The FASEB Journal, 34*(12), 16601-16621. doi:10.1096/fj.202001113R

Albanese, A., Swaney, J. M., Yun, D. H., Evans, N. B., Antonucci, J. M., Velasco, S., . . . Chung, K. (2020). Multiscale 3D Phenotyping of Human Cerebral Organoids. *Scientific reports, 10*(1), 21487. doi:10.1038/s41598-020-78130-7

Alic, I., Goh, P. A., Murray, A., Portelius, E., Gkanatsiou, E., Gough, G., . . . Nizetic, D. (2021). Patient-Specific Alzheimer-Like Pathology in Trisomy 21 Cerebral Organoids Reveals BACE2 as a Gene Dose-Sensitive AD Suppressor in Human Brain. *Molecular psychiatry, 26*(10), 5766-5788. doi:10.1038/s41380-020-0806-5

Allende, M. L., Cook, E. K., Larman, B. C., Nugent, A., Brady, J. M., Golebiowski, D., . . . Proia, R. L. (2018). Cerebral Organoids Derived From Sandhoff Disease-Induced Pluripotent Stem Cells Exhibit Impaired Neurodifferentiation. *Journal of lipid research, 59*(3), 550-563. doi:10.1194/jlr.M081323

Almenar-Queralt, A., Merkurjev, D., Kim, H. S., Navarro, M., Ma, Q., Chaves, R. S., . . . Garcia-Bassets, I. (2019). Chromatin Establishes an Immature Version of Neuronal Protocadherin Selection During the Naive-to-Primed Conversion of Pluripotent Stem Cells. *Nature genetics, 51*(12), 1691-1701. doi:10.1038/s41588-019-0526-4

Anastasaki, C., Wegscheid, M. L., Hartigan, K., Papke, J. B., Kopp, N. D., Chen, J., . . . Gutmann, D. H. (2020). Human iPSC-Derived Neurons and Cerebral Organoids Establish Differential Effects of Germline NF1 Gene Mutations. *Stem cell reports, 14*(4), 541-550.

Andersen, J., Revah, O., Miura, Y., Thom, N., Amin, N. D., Kelley, K. W., . . . Pasca, S. P. (2020). Generation of Functional Human 3D Cortico-Motor Assembloids. *Cell, 183*(7), 1913-1929 e1926. doi:10.1016/j.cell.2020.11.017

Andrews, M. G., Subramanian, L., & Kriegstein, A. R. (2020). mTOR Signaling Regulates the Morphology and Migration of Outer Radial Glia in Developing Human Cortex. *eLife, 9*. doi:10.7554/eLife.58737

Ao, Z., Cai, H., Havert, D. J., Wu, Z., Gong, Z., Beggs, J. M., . . . Guo, F. (2020). One-Stop Microfluidic Assembly of Human Brain Organoids To Model Prenatal Cannabis Exposure. *Analytical chemistry, 92*(6), 4630-4638. doi:10.1021/acs.analchem.0c00205

Arzua, T., Yan, Y., Jiang, C., Logan, S., Allison, R. L., Wells, C., . . . Bai, X. (2020). Modeling Alcohol-Induced Neurotoxicity Using Human Induced Pluripotent Stem Cell-Derived Three-Dimensional Cerebral Organoids. *Translational psychiatry, 10*(1), 347. doi:10.1038/s41398-020-01029-4

Ayala-Nunez, N. V., Follain, G., Delalande, F., Hirschler, A., Partiot, E., Hale, G. L., . . . Gaudin, R. (2019). Zika Virus Enhances Monocyte Adhesion and Transmigration Favoring Viral Dissemination to Neural Cells. *Nature communications, 10*(1), 4430. doi:10.1038/s41467-019-12408-x

Ayo-Martin, A. C., Kyrousi, C., Di Giaimo, R., & Cappello, S. (2020). GNG5 Controls the Number of Apical and Basal Progenitors and Alters Neuronal Migration During Cortical Development. *Frontiers in Molecular Biosciences, 7*, 578137. doi:10.3389/fmolb.2020.578137

Bagley, J. A., Reumann, D., Bian, S., Levi-Strauss, J., & Knoblich, J. A. (2017). Fused Cerebral Organoids Model Interactions Between Brain Regions. *Nature methods, 14*(7), 743-751. doi:10.1038/nmeth.4304

Ballabio, C., Anderle, M., Gianesello, M., Lago, C., Miele, E., Cardano, M., . . . Tiberi, L. (2020). Modeling Medulloblastoma In Vivo and With Human Cerebellar Organoids. *Nature communications, 11*(1), 583.

Bamba, Y., Kanemura, Y., Okano, H., & Yamasaki, M. (2017). Visualization of Migration of Human Cortical Neurons Generated From Induced Pluripotent Stem Cells. *Journal of neuroscience methods, 289*, 57-63. doi:10.1016/j.jneumeth.2017.07.004

Bejoy, J., Song, L., Wang, Z., Sang, Q. X., Zhou, Y., & Li, Y. (2018). Neuroprotective Activities of Heparin, Heparinase III, and Hyaluronic Acid on the Abeta42-Treated Forebrain Spheroids Derived from Human Stem Cells. *ACS biomaterials science & engineering, 4*(8), 2922-2933. doi:10.1021/acsbiomaterials.8b00021

Bejoy, J., Yuan, X., Song, L., Hua, T., Jeske, R., Sart, S., . . . Li, Y. (2019). Genomics Analysis of Metabolic Pathways of Human Stem Cell-Derived Microglia-Like Cells and the Integrated Cortical Spheroids. *Stem cells international, 2019*, 2382534. doi:10.1155/2019/2382534

Ben-Reuven, L., & Reiner, O. (2020). Toward Spatial Identities in Human Brain Organoids-on-Chip Induced by Morphogen-Soaked Beads. *Bioengineering (Basel), 7*(4). doi:10.3390/bioengineering7040164

Bendriem, R. M., Singh, S., Aleem, A. A., Antonetti, D. A., & Ross, M. E. (2019). Tight junction protein occludin regulates progenitor Self-Renewal and survival in developing cortex. *eLife, 8*. doi:10.7554/eLife.49376

Benson, C. A., Powell, H. R., Liput, M., Dinham, S., Freedman, D. A., Ignatowski, T. A., . . . Stachowiak, M. K. (2020). Immune Factor, TNFalpha, Disrupts Human Brain Organoid Development Similar to Schizophrenia-Schizophrenia Increases Developmental Vulnerability to TNFalpha. *Frontiers in cellular neuroscience, 14*, 233. doi:10.3389/fncel.2020.00233

Berger, A. A., Gil, Y., Panet, A., Weisblum, Y., Oiknine-Djian, E., Gropp, M., . . . Wolf, D. G. (2015). Transition toward Human Cytomegalovirus Susceptibility in Early Human Embryonic Stem Cell-Derived Neural Precursors. *Journal of virology, 89*(21), 11159-11164. doi:10.1128/JVI.01742-15

Bershteyn, M., Nowakowski, T. J., Pollen, A. A., Di Lullo, E., Nene, A., Wynshaw-Boris, A., & Kriegstein, A. R. (2017). Human iPSC-Derived Cerebral Organoids Model Cellular Features of Lissencephaly and Reveal Prolonged Mitosis of Outer Radial Glia. *Cell stem cell, 20*(4), 435-449 e434. doi:10.1016/j.stem.2016.12.007

Bertacchi, M., Romano, A. L., Loubat, A., Tran Mau-Them, F., Willems, M., Faivre, L., . . . Studer, M. (2020). NR2F1 regulates regional progenitor dynamics in the mouse neocortex and cortical gyrification in BBSOAS patients. *The EMBO Journal 39*(13), e104163. doi:10.15252/embj.2019104163

Bhaduri, A., Andrews, M. G., Mancia Leon, W., Jung, D., Shin, D., Allen, D., . . . Kriegstein, A. R. (2020). Cell stress in cortical organoids impairs molecular subtype specification. *Nature, 578*(7793), 142-148. doi:10.1038/s41586-020-1962-0

Bian, S., Repic, M., Guo, Z., Kavirayani, A., Burkard, T., Bagley, J. A., . . . Knoblich, J. A. (2018). Genetically engineered cerebral organoids model brain tumor formation. *Nature methods, 15*(9), 748. doi:10.1038/s41592-018-0118-8

Bilinovich, S. M., Uhl, K. L., Lewis, K., Soehnlen, X., Williams, M., Vogt, D., . . . Campbell, D. B. (2020). Integrated RNA Sequencing Reveals Epigenetic Impacts of Diesel Particulate Matter Exposure in Human Cerebral Organoids. *Developmental Neuroscience 42*(5-6), 195-207. doi:10.1159/000513536

Birey, F., Andersen, J., Makinson, C. D., Islam, S., Wei, W., Huber, N., . . . Pasca, S. P. (2017). Assembly of Functionally Integrated Human Forebrain Spheroids. *Nature, 545*(7652), 54-59. doi:10.1038/nature22330

Blair, J. D., Hockemeyer, D., & Bateup, H. S. (2018). Genetically engineered human cortical spheroid models of tuberous sclerosis. *Nature medicine, 24*(10), 1568-1578. doi:10.1038/s41591-018-0139-y

Boisvert, E. M., Means, R. E., Michaud, M., Madri, J. A., & Katz, S. G. (2019). Minocycline mitigates the effect of neonatal hypoxic insult on human brain organoids. *Cell death & disease, 10*(4), 325. doi:10.1038/s41419-019-1553-x

Boisvert, E. M., Means, R. E., Michaud, M., Thomson, J. J., Madri, J. A., & Katz, S. G. (2020). A Static Self-Directed Method for Generating Brain Organoids from Human Embryonic Stem Cells. *Journal of visualized experiments : JoVE*(157). doi:10.3791/60379

Bonnard, C., Navaratnam, N., Ghosh, K., Chan, P. W., Tan, T. T., Pomp, O., . . . Reversade, B. (2020). A loss-of-function NUAK2 mutation in humans causes anencephaly due to impaired Hippo-YAP signaling. *Journal of Experimental Medicine, 217*(12). doi:10.1084/jem.20191561

Boothe, T., Hilbert, L., Heide, M., Berninger, L., Huttner, W. B., Zaburdaev, V., . . . Rink, J. C. (2017). A tunable refractive index matching medium for live imaging cells, tissues and model organisms. *eLife, 6*. doi:10.7554/eLife.27240

Bosshard, M., Aprigliano, R., Gattiker, C., Palibrk, V., Markkanen, E., Backe, P. H., . . . van Loon, B. (2017). Impaired oxidative stress response characterizes HUWE1-promoted X-linked intellectual disability. *Scientific reports, 7*(1), 15050. doi:10.1038/s41598-017-15380-y

Boussaad, I., Obermaier, C. D., Hanss, Z., Bobbili, D. R., Bolognin, S., Glaab, E., . . . Kruger, R. (2020). A patient-based model of RNA mis-splicing uncovers treatment targets in Parkinson's disease. *Science Translational Medicine, 12*(560). doi:10.1126/scitranslmed.aau3960

Bowers, M., Liang, T., Gonzalez-Bohorquez, D., Zocher, S., Jaeger, B. N., Kovacs, W. J., . . . Jessberger, S. (2020). FASN-Dependent Lipid Metabolism Links Neurogenic Stem/Progenitor Cell Activity to Learning and Memory Deficits. *Cell stem cell, 27*(1), 98-109 e111. doi:10.1016/j.stem.2020.04.002

Brown, R. M., Rana, P., Jaeger, H. K., O'Dowd, J. M., Balemba, O. B., & Fortunato, E. A. (2019). Human Cytomegalovirus Compromises Development of Cerebral Organoids. *Journal of virology, 93*(17). doi:10.1128/JVI.00957-19

Bu, Q., Huang, Y., Li, M., Dai, Y., Fang, X., Chen, K., . . . Cen, X. (2020). Acrylamide exposure represses neuronal differentiation, induces cell apoptosis and promotes tau hyperphosphorylation in hESC-derived 3D cerebral organoids. *Food and Chemical Toxicology, 144*, 111643. doi:10.1016/j.fct.2020.111643

Buchsbaum, I. Y., Kielkowski, P., Giorgio, G., O'Neill, A. C., Di Giaimo, R., Kyrousi, C., . . . Cappello, S. (2020). ECE2 regulates neurogenesis and neuronal migration during human cortical development. *EMBO reports, 21*(5), e48204. doi:10.15252/embr.201948204

Cakir, B., Xiang, Y., Tanaka, Y., Kural, M. H., Parent, M., Kang, Y. J., . . . Park, I. H. (2019). Engineering of human brain organoids with a functional vascular-like system. *Nature methods, 16*(11), 1169-1175. doi:10.1038/s41592-019-0586-5

Camargo Ortega, G., Falk, S., Johansson, P. A., Peyre, E., Broix, L., Sahu, S. K., . . . Gotz, M. (2019). The centrosome protein AKNA regulates neurogenesis via microtubule organization. *Nature, 567*(7746), 113-117. doi:10.1038/s41586-019-0962-4

Camp, J. G., Badsha, F., Florio, M., Kanton, S., Gerber, T., Wilsch-Brauninger, M., . . . Treutlein, B. (2015). Human cerebral organoids recapitulate gene expression programs of fetal neocortex development. *the Proceedings of the National Academy of Sciences, 112*(51), 15672-15677. doi:10.1073/pnas.1520760112

Cao, Y., Hjort, M., Chen, H., Birey, F., Leal-Ortiz, S. A., Han, C. M., . . . Melosh, N. A. (2017). Nondestructive nanostraw intracellular sampling for longitudinal cell monitoring. *the Proceedings of the National Academy of Sciences, 114*(10), E1866-E1874. doi:10.1073/pnas.1615375114

Cardenas, A., Villalba, A., de Juan Romero, C., Pico, E., Kyrousi, C., Tzika, A. C., . . . Borrell, V. (2018). Evolution of Cortical Neurogenesis in Amniotes Controlled by Robo Signaling Levels. *Cell, 174*(3), 590-606 e521. doi:10.1016/j.cell.2018.06.007

Cavalcante, B. R. R., Aragao-Franca, L. S., Sampaio, G. L. A., Nonaka, C. K. V., Oliveira, M. S., Campos, G. S., . . . Soares, M. B. P. (2020). Betulinic Acid Exerts Cytoprotective Activity on Zika Virus-Infected Neural Progenitor Cells. *Frontiers in Cellular and Infectious Microbiology, 10*, 558324. doi:10.3389/fcimb.2020.558324

Cederquist, G. Y., Asciolla, J. J., Tchieu, J., Walsh, R. M., Cornacchia, D., Resh, M. D., & Studer, L. (2019). Specification of Positional Identity in Forebrain Organoids. *Nature biotechnology, 37*(4), 436-444. doi:10.1038/s41587-019-0085-3

Chen, C., Rengarajan, V., Kjar, A., & Huang, Y. (2021). A matrigel-free method to generate matured human cerebral organoids using 3D-Printed microwell arrays. *Bioactive Materials, 6*(4), 1130-1139. doi:10.1016/j.bioactmat.2020.10.003

Chen, M., Lee, H. K., Moo, L., Hanlon, E., Stein, T., & Xia, W. (2018). Common proteomic profiles of induced pluripotent stem cell-derived three-dimensional neurons and brain tissue from Alzheimer patients. *Journal of proteomics, 182*, 21-33. doi:10.1016/j.jprot.2018.04.032

Chlebanowska, P., Sulkowski, M., Skrzypek, K., Tejchman, A., Muszynska, A., Noroozi, R., & Majka, M. (2020b). Origin of the Induced Pluripotent Stem Cells Affects Their Differentiation into Dopaminergic Neurons. *International journal of molecular sciences, 21*(16). doi:10.3390/ijms21165705

Chlebanowska, P., Tejchman, A., Sulkowski, M., Skrzypek, K., & Majka, M. (2020a). Use of 3D Organoids as a Model to Study Idiopathic Form of Parkinson's Disease. *International journal of molecular sciences, 21*(3). doi:10.3390/ijms21030694

Choe, M. S., Kim, J. S., Yeo, H. C., Bae, C. M., Han, H. J., Baek, K., . . . Lee, M. Y. (2020). A simple metastatic brain cancer model using human embryonic stem cell-derived cerebral organoids. *The Federation of American Societies of Experimental Biology, 34*(12), 16464-16475. doi:10.1096/fj.202000372R

Choi, H., Kim, H. J., Yang, J., Chae, S., Lee, W., Chung, S., . . . Mook-Jung, I. (2020). Acetylation changes tau interactome to degrade tau in Alzheimer's disease animal and organoid models. *Aging cell, 19*(1), e13081. doi:10.1111/acel.13081

Chumarina, M., Russ, K., Azevedo, C., Heuer, A., Pihl, M., Collin, A., . . . Roybon, L. (2019). Cellular alterations identified in pluripotent stem cell-derived midbrain spheroids generated from a female patient with progressive external ophthalmoplegia and parkinsonism who carries a novel variation (p.Q811R) in the POLG1 gene. *Acta neuropathologica communications, 7*(1), 208. doi:10.1186/s40478-019-0863-7

Conforti, P., Besusso, D., Bocchi, V. D., Faedo, A., Cesana, E., Rossetti, G., . . . Cattaneo, E. (2018). Faulty neuronal determination and cell polarization are reverted by modulating HD early phenotypes. *the Proceeding of the National Academy of Sciences, 115*(4), E762-E771. doi:10.1073/pnas.1715865115

Cosset, E., Locatelli, M., Marteyn, A., Lescuyer, P., Dall Antonia, F., Mor, F. M., . . . Tieng, V. (2019). Human Neural Organoids for Studying Brain Cancer and Neurodegenerative Diseases. *Journal of visualized experiments : JoVE*(148). doi:10.3791/59682

Cosset, E., Martinez, Y., Preynat-Seauve, O., Lobrinus, J. A., Tapparel, C., Cordey, S., . . . Krause, K. H. (2015). Human three-dimensional engineered neural tissue reveals cellular and molecular events following cytomegalovirus infection. *Biomaterials, 53*, 296-308. doi:10.1016/j.biomaterials.2015.02.094

Coulter, M. E., Dorobantu, C. M., Lodewijk, G. A., Delalande, F., Cianferani, S., Ganesh, V. S., . . . Walsh, C. A. (2018). The ESCRT-III Protein CHMP1A Mediates Secretion of Sonic Hedgehog on a Distinctive Subtype of Extracellular Vesicles. *Cell reports, 24*(4), 973-986 e978. doi:10.1016/j.celrep.2018.06.100

Cugola, F. R., Fernandes, I. R., Russo, F. B., Freitas, B. C., Dias, J. L., Guimaraes, K. P., . . . Beltrao-Braga, P. C. (2016). The Brazilian Zika virus strain causes birth defects in experimental models. *Nature, 534*(7606), 267-271. doi:10.1038/nature18296

Cullen, D. K., Gordian-Velez, W. J., Struzyna, L. A., Jgamadze, D., Lim, J., Wofford, K. L., . . . Chen, H. I. (2019). Bundled Three-Dimensional Human Axon Tracts Derived from Brain Organoids. *iScience, 21*, 57-67. doi:10.1016/j.isci.2019.10.004

Dakic, V., Minardi Nascimento, J., Costa Sartore, R., Maciel, R. M., de Araujo, D. B., Ribeiro, S., . . . Rehen, S. K. (2017). Short Term Changes in the Proteome of Human Cerebral Organoids Induced by 5-MeO-DMT. *Scientific reports, 7*(1), 12863. doi:10.1038/s41598-017-12779-5

Dang, J., Tiwari, S. K., Agrawal, K., Hui, H., Qin, Y., & Rana, T. M. (2021). Glial Cell Diversity and Methamphetamine-Induced Neuroinflammation in Human Cerebral Organoids. *Molecular psychiatry, 26*(4), 1194-1207. doi:10.1038/s41380-020-0676-x

Dang, J., Tiwari, S. K., Lichinchi, G., Qin, Y., Patil, V. S., Eroshkin, A. M., & Rana, T. M. (2016). Zika Virus Depletes Neural Progenitors in Human Cerebral Organoids through Activation of the Innate Immune Receptor TLR3. *Cell stem cell, 19*(2), 258-265. doi:10.1016/j.stem.2016.04.014

Das, D., Li, J., Cheng, L., Franco, S., & Mahairaki, V. (2020). Human Forebrain Organoids from Induced Pluripotent Stem Cells: A Novel Approach to Model Repair of Ionizing Radiation-Induced DNA Damage in Human Neurons. *Radiation Research 194*(2), 191-198. doi:10.1667/RR15567.1

Daviaud, N., Chevalier, C., Friedel, R. H., & Zou, H. (2019). Distinct Vulnerability and Resilience of Human Neuroprogenitor Subtypes in Cerebral Organoid Model of Prenatal Hypoxic Injury. *Frontiers in cellular neuroscience, 13*, 336. doi:10.3389/fncel.2019.00336

Daviaud, N., Friedel, R. H., & Zou, H. (2018). Vascularization and Engraftment of Transplanted Human Cerebral Organoids in Mouse Cortex. *eNeuro, 5*(6). doi:10.1523/ENEURO.0219-18.2018

Depla, J. A., Sogorb-Gonzalez, M., Mulder, L. A., Heine, V. M., Konstantinova, P., van Deventer, S. J., . . . Evers, M. M. (2020). Cerebral Organoids: A Human Model for AAV Capsid Selection and Therapeutic Transgene Efficacy in the Brain. *Molecular Therapy Methods & Clinical Development, 18*, 167-175. doi:10.1016/j.omtm.2020.05.028

Dezonne, R. S., Sartore, R. C., Nascimento, J. M., Saia-Cereda, V. M., Romao, L. F., Alves-Leon, S. V., . . . Gomes, F. C. (2017). Derivation of Functional Human Astrocytes from Cerebral Organoids. *Scientific reports, 7*, 45091. doi:10.1038/srep45091

Di Matteo, F., Pipicelli, F., Kyrousi, C., Tovecci, I., Penna, E., Crispino, M., . . . Cappello, S. (2020). Cystatin B is essential for proliferation and interneuron migration in individuals with EPM1 epilepsy. *EMBO molecular medicine, 12*(6), e11419. doi:10.15252/emmm.201911419

Durens, M., Nestor, J., Williams, M., Herold, K., Niescier, R. F., Lunden, J. W., . . . Nestor, M. W. (2020). High-Throughput Screening of Human Induced Pluripotent Stem Cell-Derived Brain Organoids. *Journal of neuroscience methods, 335*, 108627. doi:10.1016/j.jneumeth.2020.108627

Dutta, A., Karanth, S. S., Bhattacharya, M., Liput, M., Augustyniak, J., Cheung, M., . . . Stachowiak, M. K. (2020). A Proof of Concept 'Phase Zero' Study of Neurodevelopment Using Brain Organoid Models with Vis/Near-Infrared Spectroscopy and Electrophysiology. *Scientific reports, 10*(1), 20987. doi:10.1038/s41598-020-77929-8

Duval, N., Vaslin, C., Barata, T. C., Frarma, Y., Contremoulins, V., Baudin, X., . . . Ribes, V. C. (2019). BMP4 Patterns Smad Activity and Generates Stereotyped Cell Fate Organization in Spinal Organoids. *Development, 146*(14). doi:10.1242/dev.175430

Eguchi, N., Sora, I., & Muguruma, K. (2018). Self-Organizing Cortex Generated From Human iPSCs with Combination of FGF2 and Ambient Oxygen. *Biochemical and biophysical research communications, 498*(4), 729-735. doi:10.1016/j.bbrc.2018.03.049

Esk, C., Lindenhofer, D., Haendeler, S., Wester, R. A., Pflug, F., Schroeder, B., . . . Knoblich, J. A. (2020). A Human Tissue Screen Identifies a Regulator of ER Secretion as a Brain-Size Determinant. *Science, 370*(6519), 935-941. doi:10.1126/science.abb5390

Eura, N., Matsui, T. K., Luginbuhl, J., Matsubayashi, M., Nanaura, H., Shiota, T., . . . Mori, E. (2020). Brainstem Organoids From Human Pluripotent Stem Cells. *Frontiers in Neuroscience, 14*, 538. doi:10.3389/fnins.2020.00538

Fair, S. R., Julian, D., Hartlaub, A. M., Pusuluri, S. T., Malik, G., Summerfied, T. L., . . . Hester, M. E. (2020). Electrophysiological Maturation of Cerebral Organoids Correlates with Dynamic Morphological and Cellular Development. *Stem cell reports, 15*(4), 855-868. doi:10.1016/j.stemcr.2020.08.017

Fiddes, I. T., Lodewijk, G. A., Mooring, M., Bosworth, C. M., Ewing, A. D., Mantalas, G. L., . . . Haussler, D. (2018). Human-Specific NOTCH2NL Genes Affect Notch Signaling and Cortical Neurogenesis. *Cell, 173*(6), 1356-1369 e1322. doi:10.1016/j.cell.2018.03.051

Field, A. R., Jacobs, F. M. J., Fiddes, I. T., Phillips, A. P. R., Reyes-Ortiz, A. M., LaMontagne, E., . . . Haussler, D. (2019). Structurally Conserved Primate LncRNAs Are Transiently Expressed during Human Cortical Differentiation and Influence Cell-Type-Specific Genes. *Stem Cell Reports 12*(2), 245-257.

Foliaki, S. T., Groveman, B. R., Yuan, J., Walters, R., Zhang, S., Tesar, P., . . . Haigh, C. L. (2020). Pathogenic Prion Protein Isoforms Are Not Present in Cerebral Organoids Generated from Asymptomatic Donors Carrying the E200K Mutation Associated with Familial Prion Disease. *Pathogens, 9*(6). doi:10.3390/pathogens9060482

Gabriel, E., & Gopalakrishnan, J. (2017). Generation of iPSC-derived Human Brain Organoids to Model Early Neurodevelopmental Disorders. *Journal of visualized experiments : JoVE*(122). doi:10.3791/55372

Gabriel, E., Ramani, A., Karow, U., Gottardo, M., Natarajan, K., Gooi, L. M., . . . Gopalakrishnan, J. (2017). Recent Zika Virus Isolates Induce Premature Differentiation of Neural Progenitors in Human Brain Organoids. *Cell stem cell, 20*(3), 397-406 e395. doi:10.1016/j.stem.2016.12.005

Gabriel, E., Wason, A., Ramani, A., Gooi, L. M., Keller, P., Pozniakovsky, A., . . . Gopalakrishnan, J. (2016). CPAP Promotes Timely Cilium Disassembly to Maintain Neural Progenitor Pool. *The EMBO journal, 35*(8), 803-819. doi:10.15252/embj.201593679

Garcez, P. P., Loiola, E. C., Madeiro da Costa, R., Higa, L. M., Trindade, P., Delvecchio, R., . . . Rehen, S. K. (2016). Zika Virus Impairs Growth in Human Neurospheres and Brain Organoids. *Science, 352*(6287), 816-818. doi:10.1126/science.aaf6116

Ghatak, S., Dolatabadi, N., Gao, R., Wu, Y., Scott, H., Trudler, D., . . . Lipton, S. A. (2021). NitroSynapsin Ameliorates Hypersynchronous Neural Network Activity in Alzheimer hiPSC Models. *Molecular psychiatry, 26*(10), 5751-5765. doi:10.1038/s41380-020-0776-7

Ghatak, S., Dolatabadi, N., Trudler, D., Zhang, X., Wu, Y., Mohata, M., . . . Lipton, S. A. (2019). Mechanisms of Hyperexcitability in Alzheimer's Disease hiPSC-Derived Neurons and Cerebral Organoids vs Isogenic Controls. *eLife, 8*. doi:10.7554/eLife.50333

Giandomenico, S. L., Mierau, S. B., Gibbons, G. M., Wenger, L. M. D., Masullo, L., Sit, T., . . . Lancaster, M. A. (2019). Cerebral Organoids at the Air-Liquid Interface Generate Diverse Nerve Tracts With Functional Output. *Nature neuroscience, 22*(4), 669-679. doi:10.1038/s41593-019-0350-2

Giandomenico, S. L., Sutcliffe, M., & Lancaster, M. A. (2021). Generation and Long-Term Culture of Advanced Cerebral Organoids for Studying Later Stages of Neural Development. *Nature protocols, 16*(2), 579-602. doi:10.1038/s41596-020-00433-w

Gomes, A. R., Fernandes, T. G., Vaz, S. H., Silva, T. P., Bekman, E. P., Xapelli, S., . . . Diogo, M. M. (2020). Modeling Rett Syndrome With Human Patient-Specific Forebrain Organoids. *Frontiers in cell and developmental biology, 8*, 610427. doi:10.3389/fcell.2020.610427

Gomez-Giro, G., Arias-Fuenzalida, J., Jarazo, J., Zeuschner, D., Ali, M., Possemis, N., . . . Schwamborn, J. C. (2019). Synapse Alterations Precede Neuronal Damage and Storage Pathology in a Human Cerebral Organoid Model of CLN3-Juvenile Neuronal Ceroid Lipofuscinosis. *Acta neuropathologica communications, 7*(1), 222. doi:10.1186/s40478-019-0871-7

Gonzalez, C., Armijo, E., Bravo-Alegria, J., Becerra-Calixto, A., Mays, C. E., & Soto, C. (2018). Modeling Amyloid Beta and Tau Pathology in Human Cerebral Organoids. *Molecular psychiatry, 23*(12), 2363-2374. doi:10.1038/s41380-018-0229-8

Goranci-Buzhala, G., Mariappan, A., Gabriel, E., Ramani, A., Ricci-Vitiani, L., Buccarelli, M., . . . Gopalakrishnan, J. (2020). Rapid and Efficient Invasion Assay of Glioblastoma in Human Brain Organoids. *Cell reports, 31*(10), 107738. doi:10.1016/j.celrep.2020.107738

Goto-Silva, L., Ayad, N. M. E., Herzog, I. L., Silva, N. P., Lamien, B., Orlande, H. R. B., . . . Rehen, S. K. (2019). Computational Fluid Dynamic Analysis of Physical Forces Playing a Role in Brain Organoid Cultures in Two Different Multiplex Platforms. *BMC developmental biology, 19*(1), 3. doi:10.1186/s12861-019-0183-y

Griffin, K., Bejoy, J., Song, L., Hua, T., Marzano, M., Jeske, R., . . . Li, Y. (2020). Human Stem Cell-derived Aggregates of Forebrain Astroglia Respond to Amyloid Beta Oligomers. *Tissue Engineering Part A, 26*(9-10), 527-542. doi:10.1089/ten.TEA.2019.0227

Groveman, B. R., Foliaki, S. T., Orru, C. D., Zanusso, G., Carroll, J. A., Race, B., & Haigh, C. L. (2019). Sporadic Creutzfeldt-Jakob Disease Prion Infection of Human Cerebral Organoids. *Acta neuropathologica communications, 7*(1), 90. doi:10.1186/s40478-019-0742-2

Ham, O., Jin, Y. B., Kim, J., & Lee, M. O. (2020). Blood Vessel Formation in Cerebral Organoids Formed From Human Embryonic Stem Cells. *Biochemical and biophysical research communications, 521*(1), 84-90. doi:10.1016/j.bbrc.2019.10.079

Harbuzariu, A., Pitts, S., Cespedes, J. C., Harp, K. O., Nti, A., Shaw, A. P., . . . Stiles, J. K. (2019). Modelling Heme-Mediated Brain Injury Associated With Cerebral Malaria in Human Brain Cortical Organoids. *Scientific reports, 9*(1), 19162. doi:10.1038/s41598-019-55631-8

Hengel, H., Bosso-Lefevre, C., Grady, G., Szenker-Ravi, E., Li, H., Pierce, S., . . . Reversade, B. (2020). Loss-of-Function Mutations in UDP-Glucose 6-Dehydrogenase Cause Recessive Developmental Epileptic Encephalopathy. *Nature communications, 11*(1), 595. doi:10.1038/s41467-020-14360-7

Holmes, D. B., & Heine, V. M. (2017b). Simplified 3D Protocol Capable of Generating Early Cortical Neuroepithelium. *Biology open, 6*(3), 402-406. doi:10.1242/bio.021725

Holmes, D. B., & Heine, V. M. (2017a). Streamlined 3D Cerebellar Differentiation Protocol with Optional 2D Modification. *Journal of visualized experiments : JoVE*(130). doi:10.3791/56888

Hor, J. H., Soh, E. S., Tan, L. Y., Lim, V. J. W., Santosa, M. M., Winanto, . . . Ng, S. Y. (2018). Cell Cycle Inhibitors Protect Motor Neurons in an Organoid Model of Spinal Muscular Atrophy. *Cell Death Dis, 9*(11), 1100. doi:10.1038/s41419-018-1081-0

Hua, T. T., Bejoy, J., Song, L., Wang, Z., Zeng, Z., Zhou, Y., . . . Sang, Q. A. (2021). Cerebellar Differentiation From Human Stem Cells Through Retinoid, Wnt, and Sonic Hedgehog Pathways. *Tissue Engineering Part A, 27*(13-14), 881-893. doi:10.1089/ten.TEA.2020.0135

Huang, J., Liu, F., Tang, H., Wu, H., Li, L., Wu, R., . . . Chen, J. (2017). Tranylcypromine Causes Neurotoxicity and Represses BHC110/LSD1 in Human-Induced Pluripotent Stem Cell-Derived Cerebral Organoids Model. *Frontiers in neurology, 8*, 626. doi:10.3389/fneur.2017.00626

Hwang, J. W., Loisel-Duwattez, J., Desterke, C., Latsis, T., Pagliaro, S., Griscelli, F., . . . Turhan, A. G. (2020). A Novel Neuronal Organoid Model Mimicking Glioblastoma (GBM) Features From Induced Pluripotent Stem Cells (iPSC). *Biochimica et Biophysica Acta General Subjects, 1864*(4), 129540. doi:10.1016/j.bbagen.2020.129540

Iefremova, V., Manikakis, G., Krefft, O., Jabali, A., Weynans, K., Wilkens, R., . . . Ladewig, J. (2017). An Organoid-Based Model of Cortical Development Identifies Non-Cell-Autonomous Defects in Wnt Signaling Contributing to Miller-Dieker Syndrome. *Cell reports, 19*(1), 50-59. doi:10.1016/j.celrep.2017.03.047

Jacob, F., Pather, S. R., Huang, W. K., Zhang, F., Wong, S. Z. H., Zhou, H., . . . Ming, G. L. (2020). Human Pluripotent Stem Cell-Derived Neural Cells and Brain Organoids Reveal SARS-CoV-2 Neurotropism Predominates in Choroid Plexus Epithelium. *Cell stem cell, 27*(6), 937-950 e939. doi:10.1016/j.stem.2020.09.016

Jamwal, V. S., Vishnu, V. V., Domreddy, A., Parekh, Y., Kumar, B. K., Chandra Shekar, P., & Singh, S. (2020). Generation of iPSC From Fetal Fibroblast Cells Obtained From an Abortus With Type-I Tri-Allelic Variants. *Stem cell research, 48*, 101963. doi:10.1016/j.scr.2020.101963

Janssens, S., Schotsaert, M., Karnik, R., Balasubramaniam, V., Dejosez, M., Meissner, A., . . . Zwaka, T. P. (2018). Zika Virus Alters DNA Methylation of Neural Genes in an Organoid Model of the Developing Human Brain. *mSystems, 3*(1). doi:10.1128/mSystems.00219-17

Janssens, S., Schotsaert, M., Manganaro, L., Dejosez, M., Simon, V., Garcia-Sastre, A., & Zwaka, T. P. (2019). FACS-Mediated Isolation of Neuronal Cell Populations From Virus-Infected Human Embryonic Stem Cell-Derived Cerebral Organoid Cultures. *Current protocols in stem cell biology, 48*(1), e65. doi:10.1002/cpsc.65

Jin, M., Pomp, O., Shinoda, T., Toba, S., Torisawa, T., Furuta, K., . . . Hirotsune, S. (2017). Katanin p80, NuMA and Cytoplasmic Dynein Cooperate to Control Microtubule Dynamics. *Scientific reports, 7*, 39902. doi:10.1038/srep39902

Jo, J., Xiao, Y., Sun, A. X., Cukuroglu, E., Tran, H. D., Goke, J., . . . Ng, H. H. (2016). Midbrain-like Organoids From Human Pluripotent Stem Cells Contain Functional Dopaminergic and Neuromelanin-Producing Neurons. *Cell stem cell, 19*(2), 248-257. doi:10.1016/j.stem.2016.07.005

Johnstone, M., Vasistha, N. A., Barbu, M. C., Dando, O., Burr, K., Christopher, E., . . . Chandran, S. (2019). Reversal of Proliferation Deficits Caused by Chromosome 16p13.11 Microduplication Through Targeting NFkappaB Signaling: an Integrated Study of Patient-Derived Neuronal Precursor Cells, Cerebral Organoids and In Vivo Brain Imaging. *Molecular psychiatry, 24*(2), 294-311. doi:10.1038/s41380-018-0292-1

Joshi, P., Bodnya, C., Rasmussen, M. L., Romero-Morales, A. I., Bright, A., & Gama, V. (2020). Modeling the Function of BAX and BAK in Early Human Brain Development Using iPSC-Derived Systems. *Cell death & disease, 11*(9), 808. doi:10.1038/s41419-020-03002-x

Kadoshima, T., Sakaguchi, H., Nakano, T., Soen, M., Ando, S., Eiraku, M., & Sasai, Y. (2013). Self-Organization of Axial Polarity, Inside-Out Layer Pattern, and Species-Specific Progenitor Dynamics in Human ES Cell-Derived Neocortex. *Proceedings of the National Academy of Sciences of the United States of America, 110*(50), 20284-20289. doi:10.1073/pnas.1315710110

Kano, M., Takanashi, M., Oyama, G., Yoritaka, A., Hatano, T., Shiba-Fukushima, K., . . . Hattori, N. (2020). Reduced Astrocytic Reactivity in Human brains and Midbrain Organoids With PRKN Mutations. *npj Parkinson's Disease 6*(1), 33. doi:10.1038/s41531-020-00137-8

Kanton, S., Boyle, M. J., He, Z., Santel, M., Weigert, A., Sanchis-Calleja, F., . . . Camp, J. G. (2019). Organoid Single-Cell Genomic Atlas Uncovers Human-Specific Features of Brain Development. *Nature, 574*(7778), 418-422. doi:10.1038/s41586-019-1654-9

Karzbrun, E., Kshirsagar, A., Cohen, S. R., Hanna, J. H., & Reiner, O. (2018). Human Brain Organoids on a Chip Reveal the Physics of Folding. *Nature physics, 14*(5), 515-522. doi:10.1038/s41567-018-0046-7

Karzbrun, E., Tshuva, R. Y., & Reiner, O. (2018). An On-Chip Method for Long-Term Growth and Real-Time Imaging of Brain Organoids. *Current protocols in cell biology, 81*(1), e62. doi:10.1002/cpcb.62

Kasai, T., Suga, H., Sakakibara, M., Ozone, C., Matsumoto, R., Kano, M., . . . Arima, H. (2020). Hypothalamic Contribution to Pituitary Functions Is Recapitulated In Vitro Using 3D-Cultured Human iPS Cells. *Cell reports, 30*(1), 18-24.e15. doi:10.1016/j.celrep.2019.12.009

Kathuria, A., Lopez-Lengowski, K., Jagtap, S. S., McPhie, D., Perlis, R. H., Cohen, B. M., & Karmacharya, R. (2020b). Transcriptomic Landscape and Functional Characterization of Induced Pluripotent Stem Cell-Derived Cerebral Organoids in Schizophrenia. *JAMA psychiatry, 77*(7), 745-754. doi:10.1001/jamapsychiatry.2020.0196

Kathuria, A., Lopez-Lengowski, K., Vater, M., McPhie, D., Cohen, B. M., & Karmacharya, R. (2020c). Transcriptome Analysis and Functional Characterization of Cerebral Organoids in Bipolar Disorder. *Genome medicine, 12*(1), 34. doi:10.1186/s13073-020-00733-6

Kathuria, A., Lopez-Lengowski, K., Watmuff, B., & Karmacharya, R. (2020a). Comparative Transcriptomic Analysis of Cerebral Organoids and Cortical Neuron Cultures Derived from Human Induced Pluripotent Stem Cells. *Stem cells and development, 29*(21), 1370-1381. doi:10.1089/scd.2020.0069

Khan, T. A., Revah, O., Gordon, A., Yoon, S. J., Krawisz, A. K., Goold, C., . . . Pasca, S. P. (2020). Neuronal Defects in a Human Cellular Model of 22q11.2 Deletion Syndrome. *Nature medicine, 26*(12), 1888-1898. doi:10.1038/s41591-020-1043-9

Kielkowski, P., Buchsbaum, I. Y., Kirsch, V. C., Bach, N. C., Drukker, M., Cappello, S., & Sieber, S. A. (2020). FICD Activity and AMPylation Remodelling Modulate Human Neurogenesis. *Nature communications, 11*(1), 517. doi:10.1038/s41467-019-14235-6

Kim, H., Park, H. J., Choi, H., Chang, Y., Park, H., Shin, J., . . . Kim, J. (2019). Modeling G2019S-LRRK2 Sporadic Parkinson's Disease in 3D Midbrain Organoids. *Stem cell reports, 12*(3), 518-531. doi:10.1016/j.stemcr.2019.01.020

Kim, H. M., Lee, S. H., Lim, J., Yoo, J., & Hwang, D. Y. (2021). The Epidermal Growth Factor Receptor Variant Type III Mutation Frequently Found in Gliomas Induces Astrogenesis in Human Cerebral Organoids. *Cell Proliferation, 54*(2), e12965. doi:10.1111/cpr.12965

Kirihara, T., Luo, Z., Chow, S. Y. A., Misawa, R., Kawada, J., Shibata, S., . . . Ikeuchi, Y. (2019). A Human Induced Pluripotent Stem Cell-Derived Tissue Model of a Cerebral Tract Connecting Two Cortical Regions. *iScience, 14*, 301-311. doi:10.1016/j.isci.2019.03.012

Kitahara, T., Sakaguchi, H., Morizane, A., Kikuchi, T., Miyamoto, S., & Takahashi, J. (2020). Axonal Extensions Along Corticospinal Tracts From Transplanted Human Cerebral Organoids. *Stem cell reports, 15*(2), 467-481. doi:10.1016/j.stemcr.2020.06.016

Klaus, J., Kanton, S., Kyrousi, C., Ayo-Martin, A. C., Di Giaimo, R., Riesenberg, S., . . . Cappello, S. (2019). Altered Neuronal Migratory Trajectories in Human Cerebral Organoids Derived From Individuals With Neuronal Heterotopia. *Nature medicine, 25*(4), 561-568. doi:10.1038/s41591-019-0371-0

Knight, G. T., Lundin, B. F., Iyer, N., Ashton, L. M., Sethares, W. A., Willett, R. M., & Ashton, R. S. (2018). Engineering Induction of Singular Neural Rosette Emergence Within hPSC-Derived Tissues. *eLife, 7*, e37549. doi:10.7554/eLife.37549

Krefft, O., Jabali, A., Iefremova, V., Koch, P., & Ladewig, J. (2018). Generation of Standardized and Reproducible Forebrain-Type Cerebral Organoids From Human Induced Pluripotent Stem Cells. *Journal of visualized experiments : JoVE*(131), e56768. doi:10.3791/56768

Krieger, T. G., Tirier, S. M., Park, J., Jechow, K., Eisemann, T., Peterziel, H., . . . Conrad, C. (2020). Modeling Glioblastoma Invasion Using Human Brain Organoids and Single-Cell Transcriptomics. *Neuro-oncology, 22*(8), 1138-1149. doi:10.1093/neuonc/noaa091

Kunze, C., Borner, K., Kienle, E., Orschmann, T., Rusha, E., Schneider, M., . . . Brack-Werner, R. (2018). Synthetic AAV/CRISPR Vectors for Blocking HIV-1 Expression in Persistently Infected Astrocytes. *Glia, 66*(2), 413-427. doi:10.1002/glia.23254

Kwak, T. H., Kang, J. H., Hali, S., Kim, J., Kim, K. P., Park, C., . . . Han, D. W. (2020). Generation of Homogeneous Midbrain Organoids With In Vivo-Like Cellular Composition Facilitates Neurotoxin-Based Parkinson's Disease Modeling. *Stem Cells, 38*(6), 727-740. doi:10.1002/stem.3163

Lancaster, M. A., Corsini, N. S., Burkard, T. R., & Knoblich, J. A. (2016). doi:10.1101/049346

Lancaster, M. A., Corsini, N. S., Wolfinger, S., Gustafson, E. H., Phillips, A. W., Burkard, T. R., . . . Knoblich, J. A. (2017). Guided Self-Organization and Cortical Plate Formation in Human Brain Organoids. *Nature biotechnology, 35*(7), 659-666. doi:10.1038/nbt.3906

Lancaster, M. A., & Knoblich, J. A. (2014). Generation of Cerebral Organoids From Human Pluripotent Stem Cells. *Nature protocols, 9*(10), 2329-2340. doi:10.1038/nprot.2014.158

Lancaster, M. A., Renner, M., Martin, C. A., Wenzel, D., Bicknell, L. S., Hurles, M. E., . . . Knoblich, J. A. (2013). Cerebral Organoids Model Human Brain Development and Microcephaly. *Nature, 501*(7467), 373-379. doi:10.1038/nature12517

Latour, Y. L., Yoon, R., Thomas, S. E., Grant, C., Li, C., Sena-Esteves, M., . . . Tifft, C. J. (2019). Human GLB1 Knockout Cerebral Organoids: A Model System for Testing AAV9-Mediated GLB1 Gene Therapy for Reducing GM1 Ganglioside Storage in GM1 Gangliosidosis. *Molecular genetics and metabolism reports, 21*, 100513. doi:10.1016/j.ymgmr.2019.100513

Lee, H. K., Velazquez Sanchez, C., Chen, M., Morin, P. J., Wells, J. M., Hanlon, E. B., & Xia, W. (2016). Three Dimensional Human Neuro-Spheroid Model of Alzheimer's Disease Based on Differentiated Induced Pluripotent Stem Cells. *PloS one, 11*(9), e0163072. doi:10.1371/journal.pone.0163072

Lee, Y. K., Hwang, S. K., Lee, S. K., Yang, J. E., Kwak, J. H., Seo, H., . . . Lee, K. (2020). Cohen Syndrome Patient iPSC-Derived Neurospheres and Forebrain-Like Glutamatergic Neurons Reveal Reduced Proliferation of Neural Progenitor Cells and Altered Expression of Synapse Genes. *Journal of Clinical Medicine, 9*(6), 1886. doi:10.3390/jcm9061886

Legnini, I., Alles, J., Karaiskos, N., Ayoub, S., & Rajewsky, N. (2019). FLAM-seq: Full-Length mRNA Sequencing Reveals Principles of Poly(A) Tail Length Control. *Nature methods, 16*(9), 879-886. doi:10.1038/s41592-019-0503-y

Li, C., Deng, Y. Q., Wang, S., Ma, F., Aliyari, R., Huang, X. Y., . . . Cheng, G. (2017). 25-Hydroxycholesterol Protects Host Against Zika Virus Infection and Its Associated Microcephaly in a Mouse Model. *Immunity, 46*(3), 446-456. doi:10.1016/j.immuni.2017.02.012

Li, R., Sun, L., Fang, A., Li, P., Wu, Q., & Wang, X. (2017b). Recapitulating Cortical Development With Organoid Culture In Vitro and Modeling Abnormal Spindle-Like (ASPM Related Primary) Microcephaly Disease. *Protein & cell, 8*(11), 823-833. doi:10.1007/s13238-017-0479-2

Li, Y., Muffat, J., Omer, A., Bosch, I., Lancaster, M. A., Sur, M., . . . Jaenisch, R. (2017a). Induction of Expansion and Folding in Human Cerebral Organoids. *Cell stem cell, 20*(3), 385-396 e383. doi:10.1016/j.stem.2016.11.017

Li, Z., Lang, Y., Sakamuru, S., Samrat, S., Trudeau, N., Kuo, L., . . . Li, H. (2020a). Methylene Blue is a Potent and Broad-Spectrum Inhibitor Against Zika Virus In Vitro and In Vivo. *Emerging Microbes & Infections, 9*(1), 2404-2416. doi:10.1080/22221751.2020.1838954

Li, Z., Xu, J., Lang, Y., Fan, X., Kuo, L., D'Brant, L., . . . Li, H. (2020b). JMX0207, a Niclosamide Derivative With Improved Pharmacokinetics, Suppresses Zika Virus Infection Both In Vitro and In Vivo. *ACS Infectious diseases, 6*(10), 2616-2628. doi:10.1021/acsinfecdis.0c00217

Lin, H., Li, Q., & Lei, Y. (2017). Three-Dimensional Tissues Using Human Pluripotent Stem Cell Spheroids as Biofabrication Building Blocks. *Biofabrication, 9*(2), 025007. doi:10.1088/1758-5090/aa663b

Lin, V. J. T., Hu, J., Zolekar, A., Yan, L. J., & Wang, Y. C. (2020). Urine Sample-Derived Cerebral Organoids Suitable for Studying Neurodevelopment and Pharmacological Responses. *Frontiers in cell and developmental biology, 8*, 304. doi:10.3389/fcell.2020.00304

Lin, Y. T., Seo, J., Gao, F., Feldman, H. M., Wen, H. L., Penney, J., . . . Tsai, L. H. (2018). APOE4 Causes Widespread Molecular and Cellular Alterations Associated With Alzheimer's Disease Phenotypes in Human iPSC-Derived Brain Cell Types. *Neuron, 98*(6), 1141-1154 e1147. doi:10.1016/j.neuron.2018.05.008

Lindborg, B. A., Brekke, J. H., Vegoe, A. L., Ulrich, C. B., Haider, K. T., Subramaniam, S., . . . O'Brien, T. D. (2016). Rapid Induction of Cerebral Organoids From Human Induced Pluripotent Stem Cells Using a Chemically Defined Hydrogel and Defined Cell Culture Medium. *Stem cells translational medicine, 5*(7), 970-979. doi:10.5966/sctm.2015-0305

Liu, F., Huang, J., & Liu, Z. (2019). Vincristine Impairs Microtubules and Causes Neurotoxicity in Cerebral Organoids. *Neuroscience, 404*, 530-540. doi:10.1016/j.neuroscience.2018.12.047

Logan, S., Arzua, T., Yan, Y., Jiang, C., Liu, X., Yu, L. K., . . . Bai, X. (2020). Dynamic Characterization of Structural, Molecular, and Electrophysiological Phenotypes of Human-Induced Pluripotent Stem Cell-Derived Cerebral Organoids, and Comparison With Fetal and Adult Gene Profiles. *Cells, 9*(5), 1301. doi:10.3390/cells9051301

Long, R. K. M., Moriarty, K. P., Cardoen, B., Gao, G., Vogl, A. W., Jean, F., . . . Nabi, I. R. (2020). Super Resolution Microscopy and Deep Learning Identify Zika Virus Reorganization of the Endoplasmic Reticulum. *Scientific reports, 10*(1), 20937. doi:10.1038/s41598-020-77170-3

Lopez-Tobon, A., Villa, C. E., Cheroni, C., Trattaro, S., Caporale, N., Conforti, P., . . . Testa, G. (2019). Human Cortical Organoids Expose a Differential Function of GSK3 on Cortical Neurogenesis. *Stem cell reports, 13*(5), 847-861. doi:10.1016/j.stemcr.2019.09.005

Luo, C., Lancaster, M. A., Castanon, R., Nery, J. R., Knoblich, J. A., & Ecker, J. R. (2016). Cerebral Organoids Recapitulate Epigenomic Signatures of the Human Fetal Brain. *Cell reports, 17*(12), 3369-3384. doi:10.1016/j.celrep.2016.12.001

Madhavan, M., Nevin, Z. S., Shick, H. E., Garrison, E., Clarkson-Paredes, C., Karl, M., . . . Tesar, P. J. (2018). Induction of Myelinating Oligodendrocytes in Human Cortical Spheroids. *Nature methods, 15*(9), 700-706. doi:10.1038/s41592-018-0081-4

Mansour, A. A., Goncalves, J. T., Bloyd, C. W., Li, H., Fernandes, S., Quang, D., . . . Gage, F. H. (2018). An In Vivo Model of Functional and Vascularized Human Brain Organoids. *Nature biotechnology, 36*(5), 432-441. doi:10.1038/nbt.4127

Marin Navarro, A., Pronk, R. J., van der Geest, A. T., Oliynyk, G., Nordgren, A., Arsenian-Henriksson, M., . . . Wilhelm, M. (2020). p53 Controls Genomic Stability and Temporal Differentiation of Human Neural Stem Cells and Affects Neural Organization in Human Brain Organoids. *Cell death & disease, 11*(1), 52. doi:10.1038/s41419-019-2208-7

Marton, R. M., Miura, Y., Sloan, S. A., Li, Q., Revah, O., Levy, R. J., . . . Pasca, S. P. (2019). Differentiation and Maturation of Oligodendrocytes in Human Three-Dimensional Neural Cultures. *Nature neuroscience, 22*(3), 484-491. doi:10.1038/s41593-018-0316-9

Marzano, M., Bejoy, J., Cheerathodi, M. R., Sun, L., York, S. B., Zhao, J., . . . Li, Y. (2019). Differential Effects of Extracellular Vesicles of Lineage-Specific Human Pluripotent Stem Cells on the Cellular Behaviors of Isogenic Cortical Spheroids. *Cells, 8*(9). doi:10.3390/cells8090993

Masselink, W., Reumann, D., Murawala, P., Pasierbek, P., Taniguchi, Y., Bonnay, F., . . . Tanaka, E. M. (2019). Broad Applicability of a Streamlined Ethyl Cinnamate-Based Clearing Procedure. *Development, 146*(3). doi:10.1242/dev.166884

Matsui, T., Nieto-Estevez, V., Kyrychenko, S., Schneider, J. W., & Hsieh, J. (2017). Retinoblastoma Protein Controls Growth, Survival and Neuronal Migration in Human Cerebral Organoids. *Development, 144*(6), 1025-1034. doi:10.1242/dev.143636

Matsui, T. K., Matsubayashi, M., Sakaguchi, Y. M., Hayashi, R. K., Zheng, C., Sugie, K., . . . Mori, E. (2018). Six-Month Cultured Cerebral Organoids From Human ES Cells Contain Matured Neural Cells. *Neuroscience letters, 670*, 75-82. doi:10.1016/j.neulet.2018.01.040

Matsumoto, R., Suga, H., Aoi, T., Bando, H., Fukuoka, H., Iguchi, G., . . . Takahashi, Y. (2020). Congenital Pituitary Hypoplasia Model Demonstrates Hypothalamic OTX2 Regulation of Pituitary Progenitor Cells. *The Journal of Clinical Investigation, 130*(2), 641-654. doi:10.1172/JCI127378

McMurtrey, R. J. (2016). Analytic Models of Oxygen and Nutrient Diffusion, Metabolism Dynamics, and Architecture Optimization in Three-Dimensional Tissue Constructs with Applications and Insights in Cerebral Organoids. *Tissue Engineering Part C, Methods, 22*(3), 221-249. doi:10.1089/ten.TEC.2015.0375

Mellios, N., Feldman, D. A., Sheridan, S. D., Ip, J. P. K., Kwok, S., Amoah, S. K., . . . Sur, M. (2018). MeCP2-Regulated miRNAs Control Early Human Neurogenesis Through Differential Effects on ERK and AKT Signaling. *Molecular psychiatry, 23*(4), 1051-1065. doi:10.1038/mp.2017.86

Meng, Q., Wang, L., Dai, R., Wang, J., Ren, Z., Liu, S., . . . Chen, C. (2020). Integrative Analyses Prioritize GNL3 as a Risk Gene for Bipolar Disorder. *Molecular psychiatry, 25*(11), 2672-2684. doi:10.1038/s41380-020-00866-5

Meyer, K., Feldman, H. M., Lu, T., Drake, D., Lim, E. T., Ling, K. H., . . . Yankner, B. A. (2019). REST and Neural Gene Network Dysregulation in iPSC Models of Alzheimer's Disease. *Cell reports, 26*(5), 1112-1127 e1119. doi:10.1016/j.celrep.2019.01.023

Miura, Y., Li, M. Y., Birey, F., Ikeda, K., Revah, O., Thete, M. V., . . . Pasca, S. P. (2020). Generation of Human Striatal Organoids and Cortico-Striatal Assembloids From Human Pluripotent Stem Cells. *Nature biotechnology, 38*(12), 1421-1430. doi:10.1038/s41587-020-00763-w

Mora-Bermúdez, F., Badsha, F., Kanton, S., Camp, J. G., Vernot, B., Köhler, K., . . . Huttner, W. B. (2016). Differences and Similarities Between Human and Chimpanzee Neural Progenitors During Cerebral Cortex Development. *eLife, 5*, e18683.

Muguruma, K., Nishiyama, A., Kawakami, H., Hashimoto, K., & Sasai, Y. (2015). Self-Organization of Polarized Cerebellar Tissue in 3D Culture of Human Pluripotent Stem Cells. *Cell reports, 10*(4), 537-550. doi:10.1016/j.celrep.2014.12.051

Nakamura, M., Shiozawa, S., Tsuboi, D., Amano, M., Watanabe, H., Maeda, S., . . . Okano, H. (2019). Pathological Progression Induced by the Frontotemporal Dementia-Associated R406W Tau Mutation in Patient-Derived iPSCs. *Stem cell reports, 13*(4), 684-699. doi:10.1016/j.stemcr.2019.08.011

Nascimento, J. M., Saia-Cereda, V. M., Sartore, R. C., da Costa, R. M., Schitine, C. S., Freitas, H. R., . . . Martins-de-Souza, D. (2019). Human Cerebral Organoids and Fetal Brain Tissue Share Proteomic Similarities. *Frontiers in cell and developmental biology, 7*, 303. doi:10.3389/fcell.2019.00303

Nassor, F., Jarray, R., Biard, D. S. F., Maiza, A., Papy-Garcia, D., Pavoni, S., . . . Yates, F. (2020). Long Term Gene Expression in Human Induced Pluripotent Stem Cells and Cerebral Organoids to Model a Neurodegenerative Disease. *Frontiers in cellular neuroscience, 14*, 14. doi:10.3389/fncel.2020.00014

Naujock, M., Speidel, A., Fischer, S., Kizner, V., Dorner-Ciossek, C., & Gillardon, F. (2020). Neuronal Differentiation of Induced Pluripotent Stem Cells from Schizophrenia Patients in Two-Dimensional and in Three-Dimensional Cultures Reveals Increased Expression of the Kv4.2 Subunit DPP6 That Contributes to Decreased Neuronal Activity. *Stem cells and development, 29*(24), 1577-1587. doi:10.1089/scd.2020.0082

Ng, A. H. M., Khoshakhlagh, P., Rojo Arias, J. E., Pasquini, G., Wang, K., Swiersy, A., . . . Church, G. M. (2021). A Comprehensive Library of Human Transcription Factors for Cell Fate Engineering. *Nature biotechnology, 39*(4), 510-519. doi:10.1038/s41587-020-0742-6

Nowakowski, T. J., Pollen, A. A., Di Lullo, E., Sandoval-Espinosa, C., Bershteyn, M., & Kriegstein, A. R. (2016). Expression Analysis Highlights AXL as a Candidate Zika Virus Entry Receptor in Neural Stem Cells. *Cell stem cell, 18*(5), 591-596. doi:10.1016/j.stem.2016.03.012

O'Neill, A. C., Kyrousi, C., Klaus, J., Leventer, R. J., Kirk, E. P., Fry, A., . . . Robertson, S. P. (2018). A Primate-Specific Isoform of PLEKHG6 Regulates Neurogenesis and Neuronal Migration. *Cell reports, 25*(10), 2729-2741 e2726. doi:10.1016/j.celrep.2018.11.029

Ogawa, J., Pao, G. M., Shokhirev, M. N., & Verma, I. M. (2018). Glioblastoma Model Using Human Cerebral Organoids. *Cell reports, 23*(4), 1220-1229. doi:10.1016/j.celrep.2018.03.105

Ormel, P. R., Vieira de Sa, R., van Bodegraven, E. J., Karst, H., Harschnitz, O., Sneeboer, M. A. M., . . . Pasterkamp, R. J. (2018). Microglia Innately Develop Within Cerebral Organoids. *Nature communications, 9*(1), 4167. doi:10.1038/s41467-018-06684-2

Osaki, T., Chow, S. Y. A., Nakanishi, Y., Hernandez, J., Kawada, J., Fujii, T., & Ikeuchi, Y. (2020). Three-Dimensional Motor Nerve Organoid Generation. *Journal of visualized experiments : JoVE*(163). doi:10.3791/61544

Otani, T., Marchetto, M. C., Gage, F. H., Simons, B. D., & Livesey, F. J. (2016). 2D and 3D Stem Cell Models of Primate Cortical Development Identify Species-Specific Differences in Progenitor Behavior Contributing to Brain Size. *Cell stem cell, 18*(4), 467-480.

Ou, M. Y., Ju, X. C., Cai, Y. J., Sun, X. Y., Wang, J. F., Fu, X. Q., . . . Luo, Z. G. (2020). Heterogeneous Nuclear Ribonucleoprotein A3 Controls Mitotic Progression of Neural Progenitors via Interaction With Cohesin. *Development, 147*(10). doi:10.1242/dev.185132

Ozone, C., Suga, H., Eiraku, M., Kadoshima, T., Yonemura, S., Takata, N., . . . Sasai, Y. (2016). Functional Anterior Pituitary Generated in Self-Organizing Culture of Human Embryonic Stem Cells. *Nature communications, 7*, 10351. doi:10.1038/ncomms10351

Pacitti, D., & Bax, B. E. (2018). The Development of an In Vitro Cerebral Organoid Model for Investigating the Pathomolecular Mechanisms Associated With the Central Nervous System Involvement in Mitochondrial Neurogastrointestinal Encephalomyopathy (MNGIE). *Nucleosides, nucleotides & nucleic acids, 37*(11), 603-617. doi:10.1080/15257770.2018.1492139

Paraiso-Luna, J., Aguareles, J., Martin, R., Ayo-Martin, A. C., Simon-Sanchez, S., Garcia-Rincon, D., . . . Galve-Roperh, I. (2020). Endocannabinoid Signalling in Stem Cells and Cerebral Organoids Drives Differentiation to Deep Layer Projection Neurons via CB1 Receptors. *Development, 147*(24). doi:10.1242/dev.192161

Parisian, A. D., Koga, T., Miki, S., Johann, P. D., Kool, M., Crawford, J. R., & Furnari, F. B. (2020). SMARCB1 Loss Interacts With Neuronal Differentiation State to Block Maturation and Impact Cell Stability. *Genes & development, 34*(19-20), 1316-1329. doi:10.1101/gad.339978.120

Parker, R. N., Cairns, D. M., Wu, W. A., Jordan, K., Guo, C., Huang, W., . . . Kaplan, D. L. (2020). Smart Material Hydrogel Transfer Devices Fabricated With Stimuli-Responsive Silk-Elastin-Like Proteins. *Advanced healthcare materials, 9*(11), e2000266. doi:10.1002/adhm.202000266

Pasca, A. M., Park, J. Y., Shin, H. W., Qi, Q., Revah, O., Krasnoff, R., . . . Pasca, S. P. (2019). Human 3D cellular Model of Hypoxic Brain Injury of Prematurity. *Nature medicine, 25*(5), 784-791. doi:10.1038/s41591-019-0436-0

Pasca, A. M., Sloan, S. A., Clarke, L. E., Tian, Y., Makinson, C. D., Huber, N., . . . Pasca, S. P. (2015). Functional Cortical Neurons and Astrocytes From Human Pluripotent Stem Cells in 3D Culture. *Nature methods, 12*(7), 671-678. doi:10.1038/nmeth.3415

Pavoni, S., Jarray, R., Nassor, F., Guyot, A. C., Cottin, S., Rontard, J., . . . Yates, F. (2018). Small-Molecule Induction of Abeta-42 Peptide Production in Human Cerebral Organoids to Model Alzheimer's Disease Associated Phenotypes. *PloS one, 13*(12), e0209150. doi:10.1371/journal.pone.0209150

Pedrosa, C., Souza, L. R. Q., Gomes, T. A., de Lima, C. V. F., Ledur, P. F., Karmirian, K., . . . Rehen, S. K. (2020). The Cyanobacterial Saxitoxin Exacerbates Neural Cell Death and Brain Malformations Induced by Zika Virus. *PLOS Neglected Tropical Diseases 14*(3), e0008060. doi:10.1371/journal.pntd.0008060

Pellegrini, L., Albecka, A., Mallery, D. L., Kellner, M. J., Paul, D., Carter, A. P., . . . Lancaster, M. A. (2020a). SARS-CoV-2 Infects the Brain Choroid Plexus and Disrupts the Blood-CSF Barrier in Human Brain Organoids. *Cell stem cell, 27*(6), 951-961 e955. doi:10.1016/j.stem.2020.10.001

Pellegrini, L., Bonfio, C., Chadwick, J., Begum, F., Skehel, M., & Lancaster, M. A. (2020b). Human CNS Barrier-Forming Organoids With Cerebrospinal Fluid Production. *Science, 369*(6500). doi:10.1126/science.aaz5626

Penna, E., Cerciello, A., Chambery, A., Russo, R., Cernilogar, F. M., Pedone, E. M., . . . Crispino, M. (2019). Cystatin B Involvement in Synapse Physiology of Rodent Brains and Human Cerebral Organoids. *Frontiers in molecular neuroscience, 12*, 195. doi:10.3389/fnmol.2019.00195

Perez-Branguli, F., Buchsbaum, I. Y., Pozner, T., Regensburger, M., Fan, W., Schray, A., . . . Winner, B. (2019). Human SPG11 Cerebral Organoids Reveal Cortical Neurogenesis Impairment. *Human molecular genetics, 28*(6), 961-971. doi:10.1093/hmg/ddy397

Perez, M. J., Ivanyuk, D., Panagiotakopoulou, V., Di Napoli, G., Kalb, S., Brunetti, D., . . . Deleidi, M. (2021). Loss of Function of the Mitochondrial Peptidase PITRM1 Induces Proteotoxic Stress and Alzheimer's Disease-Like Pathology in Human Cerebral Organoids. *Molecular psychiatry, 26*(10), 5733-5750. doi:10.1038/s41380-020-0807-4

Pettke, A., Tampere, M., Pronk, R., Wallner, O., Falk, A., Warpman Berglund, U., . . . Puumalainen, M. R. (2020). Broadly Active Antiviral Compounds Disturb Zika Virus Progeny Release Rescuing Virus-Induced Toxicity in Brain Organoids. *Viruses, 13*(1). doi:10.3390/v13010037

Pham, M. T., Pollock, K. M., Rose, M. D., Cary, W. A., Stewart, H. R., Zhou, P., . . . Waldau, B. (2018). Generation of Human Vascularized Brain Organoids. *Neuroreport, 29*(7), 588-593. doi:10.1097/WNR.0000000000001014

Pollen, A. A., Bhaduri, A., Andrews, M. G., Nowakowski, T. J., Meyerson, O. S., Mostajo-Radji, M. A., . . . Kriegstein, A. R. (2019). Establishing Cerebral Organoids as Models of Human-Specific Brain Evolution. *Cell, 176*(4), 743-756.e717 doi:10.1016/j.cell.2019.01.017

Pomeshchik, Y., Klementieva, O., Gil, J., Martinsson, I., Hansen, M. G., de Vries, T., . . . Roybon, L. (2020). Human iPSC-Derived Hippocampal Spheroids: An Innovative Tool for Stratifying Alzheimer Disease Patient-Specific Cellular Phenotypes and Developing Therapies. *Stem cell reports, 15*(1), 256-273. doi:10.1016/j.stemcr.2020.06.001

Qian, X., Jacob, F., Song, M. M., Nguyen, H. N., Song, H., & Ming, G. L. (2018). Generation of Human Brain Region-Specific Organoids Using a Miniaturized Spinning Bioreactor. *Nature protocols, 13*(3), 565-580. doi:10.1038/nprot.2017.152

Qian, X., Nguyen, H. N., Song, M. M., Hadiono, C., Ogden, S. C., Hammack, C., . . . Ming, G. L. (2016). Brain-Region-Specific Organoids Using Mini-bioreactors for Modeling ZIKV Exposure. *Cell, 165*(5), 1238-1254. doi:10.1016/j.cell.2016.04.032

Qian, X., Su, Y., Adam, C. D., Deutschmann, A. U., Pather, S. R., Goldberg, E. M., . . . Ming, G. L. (2020). Sliced Human Cortical Organoids for Modeling Distinct Cortical Layer Formation. *Cell stem cell, 26*(5), 766-781 e769. doi:10.1016/j.stem.2020.02.002

Qiao, H., Guo, M., Shang, J., Zhao, W., Wang, Z., Liu, N., . . . Chen, P. (2020). Herpes Simplex Virus Type 1 Infection Leads to Neurodevelopmental Disorder-Associated Neuropathological Changes. *PLOS Pathogens, 16*(10), e1008899. doi:10.1371/journal.ppat.1008899

Qin, L., Tiwari, A. K., Zai, C. C., Freeman, N., Zhai, D., Liu, F., . . . Muller, D. J. (2020). Regulation of Melanocortin-4-Receptor (MC4R) Expression by SNP rs17066842 is Dependent on Glucose Concentration. *European Neuropsychopharmacology, 37*, 39-48. doi:10.1016/j.euroneuro.2020.05.008

Quadrato, G., Nguyen, T., Macosko, E. Z., Sherwood, J. L., Min Yang, S., Berger, D. R., . . . Arlotta, P. (2017). Cell Diversity and Network Dynamics in Photosensitive Human Brain Organoids. *Nature, 545*(7652), 48-53. doi:10.1038/nature22047

Raja, W. K., Mungenast, A. E., Lin, Y. T., Ko, T., Abdurrob, F., Seo, J., & Tsai, L. H. (2016). Self-Organizing 3D Human Neural Tissue Derived From Induced Pluripotent Stem Cells Recapitulate Alzheimer's Disease Phenotypes. *PloS one, 11*(9), e0161969. doi:10.1371/journal.pone.0161969

Rakotoson, I., Delhomme, B., Djian, P., Deeg, A., Brunstein, M., Seebacher, C., . . . Oheim, M. (2019). Fast 3-D Imaging of Brain Organoids With a New Single-Objective Planar-Illumination Two-Photon Microscope. *Frontiers in neuroanatomy, 13*, 77. doi:10.3389/fnana.2019.00077

Ramani, A., Muller, L., Ostermann, P. N., Gabriel, E., Abida-Islam, P., Muller-Schiffmann, A., . . . Gopalakrishnan, J. (2020). SARS-CoV-2 Targets Neurons of 3D Human Brain Organoids. *The EMBO Journal 39*(20), e106230. doi:10.15252/embj.2020106230

Renner, H., Grabos, M., Becker, K. J., Kagermeier, T. E., Wu, J., Otto, M., . . . Bruder, J. M. (2020). A Fully Automated High-Throughput Workflow for 3D-Based Chemical Screening in Human Midbrain Organoids. *eLife, 9*. doi:10.7554/eLife.52904

Renner, M., Lancaster, M. A., Bian, S., Choi, H., Ku, T., Peer, A., . . . Knoblich, J. A. (2017). Self-Organized Developmental Patterning and Differentiation in Cerebral Organoids. *The EMBO Journal 36*(10), 1316-1329. doi:10.15252/embj.201694700

Rigamonti, A., Repetti, G. G., Sun, C., Price, F. D., Reny, D. C., Rapino, F., . . . Rubin, L. L. (2016). Large-Scale Production of Mature Neurons from Human Pluripotent Stem Cells in a Three-Dimensional Suspension Culture System. *Stem cell reports, 6*(6), 993-1008. doi:10.1016/j.stemcr.2016.05.010

Sacramento, C. Q., de Melo, G. R., de Freitas, C. S., Rocha, N., Hoelz, L. V., Miranda, M., . . . Souza, T. M. (2017). The Clinically Approved Antiviral Drug Sofosbuvir Inhibits Zika Virus Replication. *Scientific reports, 7*, 40920. doi:10.1038/srep40920

Sakaguchi, H., Kadoshima, T., Soen, M., Narii, N., Ishida, Y., Ohgushi, M., . . . Sasai, Y. (2015). Generation of Functional Hippocampal Neurons From Self-Organizing Human Embryonic Stem Cell-Derived Dorsomedial Telencephalic Tissue. *Nature communications, 6*, 8896. doi:10.1038/ncomms9896

Sakaguchi, H., Ozaki, Y., Ashida, T., Matsubara, T., Oishi, N., Kihara, S., & Takahashi, J. (2019). Self-Organized Synchronous Calcium Transients in a Cultured Human Neural Network Derived from Cerebral Organoids. *Stem cell reports, 13*(3), 458-473. doi:10.1016/j.stemcr.2019.05.029

Salick, M. R., Wells, M. F., Eggan, K., & Kaykas, A. (2017). Modelling Zika Virus Infection of the Developing Human Brain In Vitro Using Stem Cell Derived Cerebral Organoids. *Journal of visualized experiments : JoVE*(127). doi:10.3791/56404

Sambo, D., Li, J., Brickler, T., & Chetty, S. (2019). Transient Treatment of Human Pluripotent Stem Cells with DMSO to Promote Differentiation. *Journal of visualized experiments : JoVE*(149). doi:10.3791/59833

Sartore, R. C., Cardoso, S. C., Lages, Y. V., Paraguassu, J. M., Stelling, M. P., Madeiro da Costa, R. F., . . . Rehen, S. K. (2017). Trace Elements During Primordial Plexiform Network Formation in Human Cerebral Organoids. *PeerJ, 5*, e2927. doi:10.7717/peerj.2927

Sawada, T., Chater, T. E., Sasagawa, Y., Yoshimura, M., Fujimori-Tonou, N., Tanaka, K., . . . Kato, T. (2020). Developmental Excitation-Inhibition Imbalance Underlying Psychoses Revealed by Single-Cell Analyses of Discordant Twins-Derived Cerebral Organoids. *Molecular psychiatry, 25*(11), 2695-2711. doi:10.1038/s41380-020-0844-z

Schubert, R., Trenholm, S., Balint, K., Kosche, G., Cowan, C. S., Mohr, M. A., . . . Roska, B. (2018). Virus Stamping for Targeted Single-Cell Infection In Vitro and In Vivo. *Nature biotechnology, 36*(1), 81-88. doi:10.1038/nbt.4034

Schukking, M., Miranda, H. C., Trujillo, C. A., Negraes, P. D., & Muotri, A. R. (2018). Direct Generation of Human Cortical Organoids from Primary Cells. *Stem cells and development, 27*(22), 1549-1556. doi:10.1089/scd.2018.0112

Sen, D., Voulgaropoulos, A., Drobna, Z., & Keung, A. J. (2020). Human Cerebral Organoids Reveal Early Spatiotemporal Dynamics and Pharmacological Responses of UBE3A. *Stem cell reports, 15*(4), 845-854. doi:10.1016/j.stemcr.2020.08.006

Seo, H. H., Han, H. W., Lee, S. E., Hong, S. H., Cho, S. H., Kim, S. C., . . . Kim, J. H. (2020). Modelling Toxoplasma Gondii Infection in Human Cerebral Organoids. *Emerging Microbes & Infections, 9*(1), 1943-1954. doi:10.1080/22221751.2020.1812435

Seo, J., Kritskiy, O., Watson, L. A., Barker, S. J., Dey, D., Raja, W. K., . . . Tsai, L. H. (2017). Inhibition of p25/Cdk5 Attenuates Tauopathy in Mouse and iPSC Models of Frontotemporal Dementia. *The Journal of Neuroscience, 37*(41), 9917-9924. doi:10.1523/JNEUROSCI.0621-17.2017

Setoh, Y. X., Amarilla, A. A., Peng, N. Y. G., Griffiths, R. E., Carrera, J., Freney, M. E., . . . Khromykh, A. A. (2019). Determinants of Zika Virus Host Tropism Uncovered by Deep Mutational Scanning. *Nature Microbiology, 4*(5), 876-887.

Shakhbazau, A., Danilkovich, N., Seviaryn, I., Ermilova, T., & Kosmacheva, S. (2019). Effects of Minocycline and Rapamycin in Gamma-Irradiated Human Embryonic Stem Cells-Derived Cerebral Organoids. *Molecular biology reports, 46*(1), 1343-1348. doi:10.1007/s11033-018-4552-6

Shi, Y., Sun, L., Wang, M., Liu, J., Zhong, S., Li, R., . . . Wang, X. (2020). Vascularized Human Cortical Organoids (vOrganoids) Model Cortical Development In Vivo. *PLOS Biology, 18*(5), e3000705. doi:10.1371/journal.pbio.3000705

Silva, T. P., Fernandes, T. G., Nogueira, D. E. S., Rodrigues, C. A. V., Bekman, E. P., Hashimura, Y., . . . Cabral, J. M. S. (2020). Scalable Generation of Mature Cerebellar Organoids from Human Pluripotent Stem Cells and Characterization by Immunostaining. *Journal of visualized experiments : JoVE*(160). doi:10.3791/61143

Sison, S. L., O'Brien, B. S., Johnson, A. J., Seminary, E. R., Terhune, S. S., & Ebert, A. D. (2019). Human Cytomegalovirus Disruption of Calcium Signaling in Neural Progenitor Cells and Organoids. *Journal of virology, 93*(17). doi:10.1128/JVI.00954-19

Sivitilli, A. A., Gosio, J. T., Ghoshal, B., Evstratova, A., Trcka, D., Ghiasi, P., . . . Attisano, L. (2020). Robust Production of Uniform Human Cerebral Organoids From Pluripotent Stem Cells. *Life science alliance, 3*(5). doi:10.26508/lsa.202000707

Skardal, A., Aleman, J., Forsythe, S., Rajan, S., Murphy, S., Devarasetty, M., . . . Atala, A. (2020). Drug Compound Screening in Single and Integrated Multi-Organoid Body-on-a-Chip Systems. *Biofabrication, 12*(2), 025017. doi:10.1088/1758-5090/ab6d36

Sloan, S. A., Andersen, J., Pasca, A. M., Birey, F., & Pasca, S. P. (2018). Generation and Assembly of Human Brain Region-Specific Three-Dimensional Cultures. *Nature protocols, 13*(9), 2062-2085. doi:10.1038/s41596-018-0032-7

Sloan, S. A., Darmanis, S., Huber, N., Khan, T. A., Birey, F., Caneda, C., . . . Pasca, S. P. (2017). Human Astrocyte Maturation Captured in 3D Cerebral Cortical Spheroids Derived from Pluripotent Stem Cells. *Neuron, 95*(4), 779-790 e776. doi:10.1016/j.neuron.2017.07.035

Son, M. Y., Sim, H., Son, Y. S., Jung, K. B., Lee, M. O., Oh, J. H., . . . Kim, J. (2017). Distinctive Genomic Signature of Neural and Intestinal Organoids From Familial Parkinson's Disease Patient-Derived Induced Pluripotent Stem Cells. *Neuropathology and Applied Neurobiology 43*(7), 584-603. doi:10.1111/nan.12396

Song, L., Tsai, A. C., Yuan, X., Bejoy, J., Sart, S., Ma, T., & Li, Y. (2018). Neural Differentiation of Spheroids Derived from Human Induced Pluripotent Stem Cells-Mesenchymal Stem Cells Coculture. *Tissue Engineering Part A, 24*(11-12), 915-929. doi:10.1089/ten.TEA.2017.0403

Song, L., Yuan, X., Jones, Z., Vied, C., Miao, Y., Marzano, M., . . . Li, Y. (2019). Functionalization of Brain Region-specific Spheroids with Isogenic Microglia-like Cells. *Scientific reports, 9*(1), 11055. doi:10.1038/s41598-019-47444-6

Srikanth, P., Lagomarsino, V. N., Muratore, C. R., Ryu, S. C., He, A., Taylor, W. M., . . . Young-Pearse, T. L. (2018). Shared Effects of DISC1 Disruption and Elevated WNT Signaling in Human Cerebral Organoids. *Translational psychiatry, 8*(1), 77. doi:10.1038/s41398-018-0122-x

Sriram, D., Chintala, R., Parthasaradhi, B. V. V., Nayak, S. C., Mariappan, I., & Radha, V. (2020). Expression of a Novel Brain Specific Isoform of C3G is Regulated During Development. *Scientific reports, 10*(1), 18838. doi:10.1038/s41598-020-75813-z

Stachowiak, E. K., Benson, C. A., Narla, S. T., Dimitri, A., Chuye, L. E. B., Dhiman, S., . . . Stachowiak, M. K. (2017). Cerebral Organoids Reveal Early Cortical Maldevelopment in Schizophrenia-Computational Anatomy and Genomics, Role of FGFR1. *Translational psychiatry, 7*(11), 6. doi:10.1038/s41398-017-0054-x

Subramanian, L., Bershteyn, M., Paredes, M. F., & Kriegstein, A. R. (2017). Dynamic Behaviour of Human Neuroepithelial Cells in the Developing Forebrain. *Nature communications, 8*, 14167. doi:10.1038/ncomms14167

Sun, A. X., Yuan, Q., Fukuda, M., Yu, W., Yan, H., Lim, G. G. Y., . . . Je, H. S. (2019). Potassium Channel Dysfunction in Human Neuronal Models of Angelman Syndrome. *Science, 366*(6472), 1486-1492. doi:10.1126/science.aav5386

Sun, G., Chiuppesi, F., Chen, X., Wang, C., Tian, E., Nguyen, J., . . . Shi, Y. (2020). Modeling Human Cytomegalovirus-Induced Microcephaly in Human iPSC-Derived Brain Organoids. *Cell Reports Medicine, 1*(1), 100002. doi:10.1016/j.xcrm.2020.100002

Sutcliffe, M., & Lancaster, M. A. (2019). A Simple Method of Generating 3D Brain Organoids Using Standard Laboratory Equipment. *Methods in Molecular Biology, 1576*, 1-12. doi:10.1007/7651_2017_2

Tejchman, A., Znoj, A., Chlebanowska, P., Fraczek-Szczypta, A., & Majka, M. (2020). Carbon Fibers as a New Type of Scaffold for Midbrain Organoid Development. *International journal of molecular sciences, 21*(17). doi:10.3390/ijms21175959

Thomas, C. A., Tejwani, L., Trujillo, C. A., Negraes, P. D., Herai, R. H., Mesci, P., . . . Muotri, A. R. (2017). Modeling of TREX1-Dependent Autoimmune Disease using Human Stem Cells Highlights L1 Accumulation as a Source of Neuroinflammation. *Cell stem cell, 21*(3), 319-331 e318. doi:10.1016/j.stem.2017.07.009

Tieng, V., Stoppini, L., Villy, S., Fathi, M., Dubois-Dauphin, M., & Krause, K. H. (2014). Engineering of Midbrain Organoids Containing Long-Lived Dopaminergic Neurons. *Stem cells and development, 23*(13), 1535-1547. doi:10.1089/scd.2013.0442

Tomaskovic-Crook, E., & Crook, J. M. (2019). Clinically Amendable, Defined, and Rapid Induction of Human Brain Organoids from Induced Pluripotent Stem Cells. *Methods in Molecular Biology, 1576*, 13-22. doi:10.1007/7651_2017_95

Tournier, N., Goutal, S., Mairinger, S., Hernandez-Lozano, I., Filip, T., Sauberer, M., . . . Langer, O. (2021). Complete Inhibition of ABCB1 and ABCG2 at the Blood-Brain Barrier by Co-Infusion of Erlotinib and Tariquidar to Improve Brain Delivery of the Model ABCB1/ABCG2 Substrate [(11)C]Erlotinib. *Journal of Cerebral Blood Flow & Metabolism, 41*(7), 1634-1646. doi:10.1177/0271678X20965500

Trevino, A. E., Sinnott-Armstrong, N., Andersen, J., Yoon, S. J., Huber, N., Pritchard, J. K., . . . Pasca, S. P. (2020). Chromatin Accessibility Dynamics in a Model of Human Forebrain Development. *Science, 367*(6476). doi:10.1126/science.aay1645

Trujillo, C. A., Adams, J. W., Negraes, P. D., Carromeu, C., Tejwani, L., Acab, A., . . . Muotri, A. R. (2021). Pharmacological Reversal of Synaptic and Network Pathology in Human MECP2-KO Neurons and Cortical Organoids. *EMBO molecular medicine, 13*(1), e12523. doi:10.15252/emmm.202012523

Trujillo, C. A., Gao, R., Negraes, P. D., Gu, J., Buchanan, J., Preissl, S., . . . Muotri, A. R. (2019). Complex Oscillatory Waves Emerging From Cortical Organoids Model Early Human Brain Network Development. *Cell stem cell, 25*(4), 558-569 e557. doi:10.1016/j.stem.2019.08.002

Turelli, P., Playfoot, C., Grun, D., Raclot, C., Pontis, J., Coudray, A., . . . Trono, D. (2020). Primate-restricted KRAB zinc finger proteins and target retrotransposons control gene expression in human neurons. *Sci Adv, 6*(35), eaba3200. doi:10.1126/sciadv.aba3200

Velasco, S., Kedaigle, A. J., Simmons, S. K., Nash, A., Rocha, M., Quadrato, G., . . . Arlotta, P. (2019). Individual Brain Organoids Reproducibly Form Cell Diversity of the Human Cerebral Cortex. *Nature, 570*(7762), 523-527. doi:10.1038/s41586-019-1289-x

Villar-Vesga, J., Henao-Restrepo, J., Voshart, D. C., Aguillon, D., Villegas, A., Castano, D., . . . Posada-Duque, R. (2020). Differential Profile of Systemic Extracellular Vesicles From Sporadic and Familial Alzheimer's Disease Leads to Neuroglial and Endothelial Cell Degeneration. *Frontiers in Aging Neuroscience, 12*, 587989. doi:10.3389/fnagi.2020.587989

Wang, L., Hou, S., & Han, Y. G. (2016). Hedgehog Signaling Promotes Basal Progenitor Expansion and the Growth and Folding of the Neocortex. *Nature neuroscience, 19*(7), 888-896. doi:10.1038/nn.4307

Wang, L., Li, Z., Sievert, D., Smith, D. E. C., Mendes, M. I., Chen, D. Y., . . . Gleeson, J. G. (2020b). Loss of NARS1 Impairs Progenitor Proliferation in Cortical Brain Organoids and Leads to Microcephaly. *Nature communications, 11*(1), 4038. doi:10.1038/s41467-020-17454-4

Wang, Q., Dong, X., Hu, T., Qu, C., Lu, J., Zhou, Y., . . . Pei, G. (2021). Constitutive Activity of Serotonin Receptor 6 Regulates Human Cerebral Organoids Formation and Depression-like Behaviors. *Stem cell reports, 16*(1), 75-88. doi:10.1016/j.stemcr.2020.11.015

Wang, Q., Dong, X., Lu, J., Hu, T., & Pei, G. (2020a). Constitutive Activity of a G Protein-Coupled Receptor, DRD1, Contributes to Human Cerebral Organoid Formation. *Stem Cells, 38*(5), 653-665. doi:10.1002/stem.3156

Wang, S. N., Wang, Z., Xu, T. Y., Cheng, M. H., Li, W. L., & Miao, C. Y. (2020c). Cerebral Organoids Repair Ischemic Stroke Brain Injury. *Translational stroke research, 11*(5), 983-1000. doi:10.1007/s12975-019-00773-0

Wang, Y., Wang, L., Zhu, Y., & Qin, J. (2018). Human Brain Organoid-on-a-Chip to Model Prenatal Nicotine Exposure. *Lab on a chip, 18*(6), 851-860. doi:10.1039/c7lc01084b

Wang, Z., Wang, S. N., Xu, T. Y., Hong, C., Cheng, M. H., Zhu, P. X., . . . Miao, C. Y. (2020a). Cerebral Organoids Transplantation Improves Neurological Motor Function in Rat Brain Injury. *CNS Neuroscience & Therapeutics 26*(7), 682-697. doi:10.1111/cns.13286

Watanabe, M., Buth, J. E., Vishlaghi, N., de la Torre-Ubieta, L., Taxidis, J., Khakh, B. S., . . . Novitch, B. G. (2017). Self-Organized Cerebral Organoids With Human-Specific Features Predict Effective Drugs to Combat Zika Virus Infection. *Cell reports, 21*(2), 517-532. doi:10.1016/j.celrep.2017.09.047

Wells, M. F., Salick, M. R., Wiskow, O., Ho, D. J., Worringer, K. A., Ihry, R. J., . . . Eggan, K. (2016). Genetic Ablation of AXL Does Not Protect Human Neural Progenitor Cells and Cerebral Organoids from Zika Virus Infection. *Cell stem cell, 19*(6), 703-708. doi:10.1016/j.stem.2016.11.011

Wilpert, N. M., Krueger, M., Opitz, R., Sebinger, D., Paisdzior, S., Mages, B., . . . Biebermann, H. (2020). Spatiotemporal Changes of Cerebral Monocarboxylate Transporter 8 Expression. *Thyroid, 30*(9), 1366-1383. doi:10.1089/thy.2019.0544

Wilson, E., Knudson, W., & Newell-Litwa, K. (2020). Hyaluronan Regulates Synapse Formation and Function in Developing Neural Networks. *Scientific reports, 10*(1), 16459. doi:10.1038/s41598-020-73177-y

Wilson, E., Rudisill, T., Kirk, B., Johnson, C., Kemper, P., & Newell-Litwa, K. (2020). Cytoskeletal Regulation of Synaptogenesis in a Model of Human Fetal Brain Development. *Journal of Neuroscience Research, 98*(11), 2148-2165. doi:10.1002/jnr.24692

Winanto, Khong, Z. J., Soh, B. S., Fan, Y., & Ng, S. Y. (2020). Organoid Cultures of MELAS Neural Cells Reveal Hyperactive Notch Signaling that Impacts Neurodevelopment. *Cell death & disease, 11*(3), 182. doi:10.1038/s41419-020-2383-6

Winkler, C. W., Woods, T. A., Groveman, B. R., Carmody, A. B., Speranza, E. E., Martens, C. A., . . . Peterson, K. E. (2019). Neuronal Maturation Reduces the Type I IFN Response to Orthobunyavirus Infection and Leads to Increased Apoptosis of Human Neurons. *Journal of neuroinflammation, 16*(1), 229. doi:10.1186/s12974-019-1614-1

Wu, W., Yao, H., Dwivedi, I., Negraes, P. D., Zhao, H. W., Wang, J., . . . Haddad, G. G. (2020). Methadone Suppresses Neuronal Function and Maturation in Human Cortical Organoids. *Frontiers in Neuroscience, 14*, 593248. doi:10.3389/fnins.2020.593248

Xiang, Y., Cakir, B., & Park, I. H. (2020). Generation of Regionally Specified Human Brain Organoids Resembling Thalamus Development. *STAR Protocols, 1*(1). doi:10.1016/j.xpro.2019.100001

Xiang, Y., Tanaka, Y., Cakir, B., Patterson, B., Kim, K. Y., Sun, P., . . . Park, I. H. (2019). hESC-Derived Thalamic Organoids Form Reciprocal Projections When Fused with Cortical Organoids. *Cell stem cell, 24*(3), 487-497 e487. doi:10.1016/j.stem.2018.12.015

Xiang, Y., Tanaka, Y., Patterson, B., Kang, Y. J., Govindaiah, G., Roselaar, N., . . . Park, I. H. (2017). Fusion of Regionally Specified hPSC-Derived Organoids Models Human Brain Development and Interneuron Migration. *Cell stem cell, 21*(3), 383-398 e387. doi:10.1016/j.stem.2017.07.007

Xiang, Y., Yoshiaki, T., Patterson, B., Cakir, B., Kim, K. Y., Cho, Y. S., & Park, I. H. (2018). Generation and Fusion of Human Cortical and Medial Ganglionic Eminence Brain Organoids. *Current protocols in stem cell biology, 47*(1), e61. doi:10.1002/cpsc.61

Xu, M., Lee, E. M., Wen, Z., Cheng, Y., Huang, W. K., Qian, X., . . . Tang, H. (2016). Identification of Small-Molecule Inhibitors of Zika Virus Infection and Induced Neural Cell Death via a Drug Repurposing Screen. *Nature medicine, 22*(10), 1101-1107. doi:10.1038/nm.4184

Xu, Y. P., Qiu, Y., Zhang, B., Chen, G., Chen, Q., Wang, M., . . . Qin, C. F. (2019). Zika Virus Infection Induces RNAi-Mediated Antiviral Immunity in Human Neural Progenitors and Brain Organoids. *Cell research, 29*(4), 265-273. doi:10.1038/s41422-019-0152-9

Yakoub, A. M., & Sadek, M. (2018). Development and Characterization of Human Cerebral Organoids: An Optimized Protocol. *Cell transplantation, 27*(3), 393-406. doi:10.1177/0963689717752946

Yakoub, A. M., & Sadek, M. (2019). Analysis of Synapses in Cerebral Organoids. *Cell transplantation, 28*(9-10), 1173-1182. doi:10.1177/0963689718822811

Yan, Y., Song, L., Bejoy, J., Zhao, J., Kanekiyo, T., Bu, G., . . . Li, Y. (2018a). Modeling Neurodegenerative Microenvironment Using Cortical Organoids Derived from Human Stem Cells. *Tissue Engineering Part A, 24*(13-14), 1125-1137. doi:10.1089/ten.TEA.2017.0423

Yan, Y., Song, L., Madinya, J., Ma, T., & Li, Y. (2018b). Derivation of Cortical Spheroids From Human Induced Pluripotent Stem Cells in a Suspension Bioreactor. *Tissue Engineering Part A, 24*(5-6), 418-431. doi:10.1089/ten.TEA.2016.0400

Yang, X., Xu, B., Mulvey, B., Evans, M., Jordan, S., Wang, Y. D., . . . Peng, J. C. (2019). Differentiation of Human Pluripotent Stem Cells into Neurons or Cortical Organoids Requires Transcriptional Co-Regulation by UTX and 53BP1. *Nature neuroscience, 22*(3), 362-373. doi:10.1038/s41593-018-0328-5

Yao, H., Wu, W., Cerf, I., Zhao, H. W., Wang, J., Negraes, P. D., . . . Haddad, G. G. (2020). Methadone Interrupts Neural Growth and Function in Human Cortical Organoids. *Stem cell research, 49*, 102065. doi:10.1016/j.scr.2020.102065

Ye, F., Kang, E., Yu, C., Qian, X., Jacob, F., Yu, C., . . . Zhang, M. (2017). DISC1 Regulates Neurogenesis via Modulating Kinetochore Attachment of Ndel1/Nde1 during Mitosis. *Neuron, 96*(5), 1041-1054 e1045. doi:10.1016/j.neuron.2017.10.010

Yi, S. A., Nam, K. H., Yun, J., Gim, D., Joe, D., Kim, Y. H., . . . Lee, J. (2020). Infection of Brain Organoids and 2D Cortical Neurons with SARS-CoV-2 Pseudovirus. *Viruses, 12*(9). doi:10.3390/v12091004

Yin, J., & VanDongen, A. M. (2021). Enhanced Neuronal Activity and Asynchronous Calcium Transients Revealed in a 3D Organoid Model of Alzheimer's Disease. *ACS biomaterials science & engineering, 7*(1), 254-264. doi:10.1021/acsbiomaterials.0c01583

Yoon, K. J., Ringeling, F. R., Vissers, C., Jacob, F., Pokrass, M., Jimenez-Cyrus, D., . . . Song, H. (2017). Temporal Control of Mammalian Cortical Neurogenesis by m(6)A Methylation. *Cell, 171*(4), 877-889 e817. doi:10.1016/j.cell.2017.09.003

Yoon, K. J., Song, G., Qian, X., Pan, J., Xu, D., Rho, H. S., . . . Ming, G. L. (2017). Zika-Virus-Encoded NS2A Disrupts Mammalian Cortical Neurogenesis by Degrading Adherens Junction Proteins. *Cell stem cell, 21*(3), 349-358 e346. doi:10.1016/j.stem.2017.07.014

Yoon, S. J., Elahi, L. S., Pasca, A. M., Marton, R. M., Gordon, A., Revah, O., . . . Pasca, S. P. (2019). Reliability of Human Cortical Organoid Generation. *Nature methods, 16*(1), 75-78. doi:10.1038/s41592-018-0255-0

Zabolocki, M., McCormack, K., van den Hurk, M., Milky, B., Shoubridge, A. P., Adams, R., . . . Bardy, C. (2020). BrainPhys Neuronal Medium Optimized for Imaging and Optogenetics In Vitro. *Nature communications, 11*(1), 5550. doi:10.1038/s41467-020-19275-x

Zafeiriou, M. P., Bao, G., Hudson, J., Halder, R., Blenkle, A., Schreiber, M. K., . . . Zimmermann, W. H. (2020). Developmental GABA Polarity Switch and Neuronal Plasticity in Bioengineered Neuronal Organoids. *Nature communications, 11*(1), 3791. doi:10.1038/s41467-020-17521-w

Zhang, B., He, Y., Xu, Y., Mo, F., Mi, T., Shen, Q. S., . . . Zhou, G. (2018). Differential Antiviral Immunity to Japanese Encephalitis Virus in Developing Cortical Organoids. *Cell Death & Disease 9*(7), 719. doi:10.1038/s41419-018-0763-y

Zhang, B. Z., Chu, H., Han, S., Shuai, H., Deng, J., Hu, Y. F., . . . Huang, J. D. (2020b). SARS-CoV-2 Infects Human Neural Progenitor Cells and Brain Organoids. *Cell research, 30*(10), 928-931. doi:10.1038/s41422-020-0390-x

Zhang, I., Lepine, P., Han, C., Lacalle-Aurioles, M., Chen, C. X., Haag, R., . . . Maysinger, D. (2020a). Nanotherapeutic Modulation of Human Neural Cells and Glioblastoma in Organoids and Monocultures. *Cells, 9*(11). doi:10.3390/cells9112434

Zhang, W., Ma, L., Yang, M., Shao, Q., Xu, J., Lu, Z., . . . Chen, J. F. (2020c). Cerebral Organoid and Mouse Models Reveal a RAB39b-PI3K-mTOR Pathway-Dependent Dysregulation of Cortical Development Leading to Macrocephaly/Autism Phenotypes. *Genes & development, 34*(7-8), 580-597. doi:10.1101/gad.332494.119

Zhang, W., Yang, S. L., Yang, M., Herrlinger, S., Shao, Q., Collar, J. L., . . . Chen, J. F. (2019). Modeling Microcephaly With Cerebral Organoids Reveals a WDR62-CEP170-KIF2A Pathway Promoting Cilium Disassembly in Neural Progenitors. *Nature communications, 10*(1), 2612. doi:10.1038/s41467-019-10497-2

Zhao, J., Fu, Y., Yamazaki, Y., Ren, Y., Davis, M. D., Liu, C. C., . . . Bu, G. (2020b). APOE4 Exacerbates Synapse Loss and Neurodegeneration in Alzheimer's Disease Patient iPSC-Derived Cerebral Organoids. *Nature communications, 11*(1), 5540. doi:10.1038/s41467-020-19264-0

Zhao, J., Ye, Z., Yang, J., Zhang, Q., Shan, W., Wang, X., . . . Ren, L. (2020a). Nanocage Encapsulation Improves Antiepileptic Efficiency of Phenytoin. *Biomaterials, 240*, 119849. doi:10.1016/j.biomaterials.2020.119849

Zheng, X., Zhang, L., Kuang, Y., Venkataramani, V., Jin, F., Hein, K., . . . Doeppner, T. R. (2021). Extracellular Vesicles Derived from Neural Progenitor Cells--a Preclinical Evaluation for Stroke Treatment in Mice. *Translational stroke research, 12*(1), 185-203. doi:10.1007/s12975-020-00814-z

Zhou, T., Tan, L., Cederquist, G. Y., Fan, Y., Hartley, B. J., Mukherjee, S., . . . Chen, S. (2017). High-Content Screening in hPSC-Neural Progenitors Identifies Drug Candidates that Inhibit Zika Virus Infection in Fetal-like Organoids and Adult Brain. *Cell stem cell, 21*(2), 274-283 e275. doi:10.1016/j.stem.2017.06.017

Zhu, Y., Wang, L., Yin, F., Yu, Y., Wang, Y., Liu, H., . . . Qin, J. (2017a). A Hollow Fiber System for Simple Generation of Human Brain Organoids. *Integrative Biology, 9*(9), 774-781. doi:10.1039/c7ib00080d

Zhu, Y., Wang, L., Yin, F., Yu, Y., Wang, Y., Shepard, M. J., . . . Qin, J. (2017c). Probing Impaired Neurogenesis in Human Brain Organoids Exposed to Alcohol. *Integrative Biology, 9*(12), 968-978. doi:10.1039/c7ib00105c

Zhu, Y., Wang, L., Yu, H., Yin, F., Wang, Y., Liu, H., . . . Qin, J. (2017b). In situ Generation of Human Brain Organoids on a Micropillar Array. *Lab on a chip, 17*(17), 2941-2950. doi:10.1039/c7lc00682a
